# Supplementary material for: Intrinsic signal amplification by type III CRISPR-Cas systems provides a sequence-specific SARS-CoV-2 diagnostic
Source: Cell Rep Med. 2021 May 27;2(6):100319. doi: 10.1016/j.xcrm.2021.100319 (PMC8157118; doi:10.1016/j.xcrm.2021.100319)
Supplement: Document S1. Figures S1–S6 and Tables S1–S5 [file mmc1.pdf]

Cell Reports Medicine, Volume *2*

Supplemental information

Intrinsic signal amplification by type III CRISPR-Cas systems provides a sequence-specific SARS-CoV-2 diagnostic

Andrew Santiago-Frangos, Laina N. Hall, Anna Nemudraia, Artem Nemudryi, Pushya Krishna, Tanner Wiegand, Royce A. Wilkinson, Deann T. Snyder, Jodi F. Hedges, Calvin Cicha, Helen H. Lee, Ava Graham, Mark A. Jutila, Matthew P. Taylor, and Blake Wiedenheft

Supplementary Information

Intrinsic Signal Amplification by Type-III CRISPR-Cas Systems Provides a Sequence-Specific Viral Diagnostic

Andrew Santiago-Frangos, Laina N. Hall, Anna Nemudraia, Artem Nemudryi, Pushya Krishna, Tanner Wiegand, Royce A. Wilkinson, Deann T. Snyder, Jodi F. Hedges, Calvin Cicha, Helen H. Lee, Ava Graham, Mark A. Jutila, Matthew P. Taylor, and Blake Wiedenheft*

Department of Microbiology and Immunology, Montana State University, Bozeman, MT 59717, USA

*Correspondence to BW: bwiedenheft@gmail.com

Supplementary Fig. 1

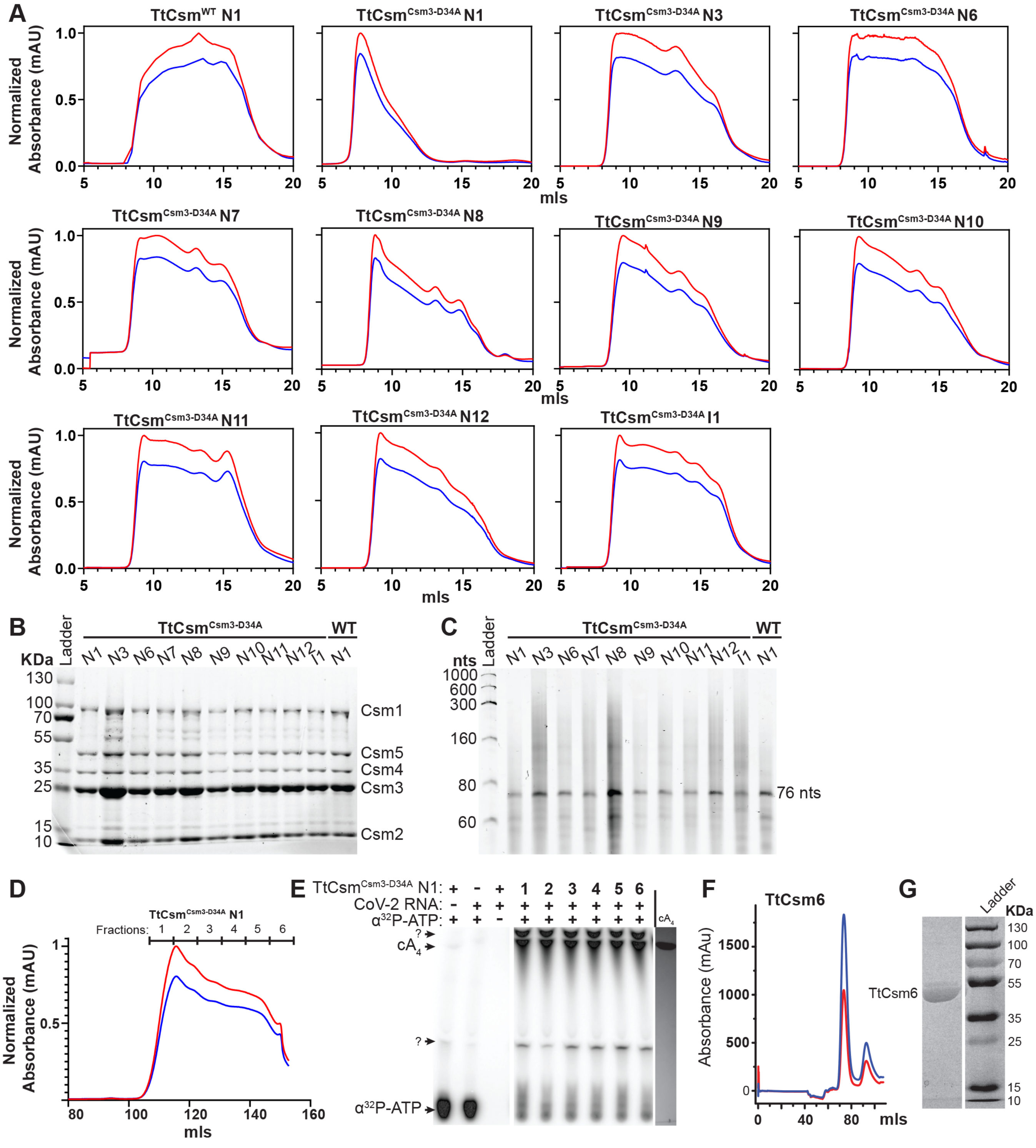

Figure S1. Purification of TtCsm complexes and TtCsm6 nuclease. Related to Figures 1-3. (**A**) Size exclusion chromatography (SEC) profiles of $TtCsm^{WT}$ and $TtCsm^{Csm3\text{-}D34A}$ complexes loaded with different crRNA guides. SEC performed using a Superose 6 Increase 10/300 GL size-exclusion column (Cytiva). Normalized absorbance (mAU) was measured at 260nm (red) and 280nm (blue). Fractions 9 up to16 of each SEC purification were collected, concentrated, and stored at -80°C. (**B**) The fractions were combined, concentrated and run on an SDS-PAGE. All five Csm proteins are present and the intensities of each band correspond with our understanding of the protein stoichiometry of the assembled TtCsm complex. (**C**) RNA was isolated from the pooled and concentrated SEC fractions. Denaturing urea polyacrylamide gel of nucleic acids associated with each TtCsm complex. The full-length crRNA intermediate is expected to be 76 nucleotides (nts) long. (**D**) SEC profile of $TtCsm^{Csm3\text{-}D34A}$ N1 complex. Six successive fractions, representing the entire peak, were collected concentrated and stored separately. (**E**) Sequence-specific activation of Cas10 was estimated for each of the six fractions in panel D. ^{32}P-ATP polymerization was measured using thin-layer chromatography (TLC). 500 nM $TtCsm^{Csm3\text{-}D34A}$ N1 complex was incubated with 10^{10} copies of target RNA, 50 µM ATP and 10 nM α^{32}P-ATP, at 60°C for 1 hour. Nucleic acids were phenol-chloroform extracted from each reaction and spotted on a silica gel TLC plate coated with fluorescent indicator F254, developed in solvent (0.2 M ammonium bicarbonate pH 9.3, 70% ethanol, 30% water). An unlabeled cA_4 standard (Axxora) was run on the same TLC plate, in a parallel lane, and was visualized by illumination with a handheld shortwave (254 nm) UV lamp (Analytik Jena) and a Galaxy S9 phone (Samsung). One of the two major ^{32}P-labelled products generated by target RNA-bound TtCsm complex migrates similarly to the cA_4 standard. All TtCsm complexes polymerize similar amounts of α^{32}P-ATP (bottom band) into similar ratios of similarly migrating products (top bands). (**F**) SEC profile of TtCsm6 ancillary nuclease purified on a Superdex 200 26/600 size-exclusion column (Cytiva). (**G**) SDS-PAGE gel image of purified TtCsm6.

Supplementary Fig. 2

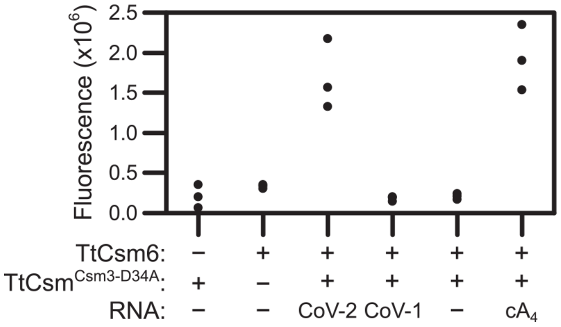

Figure S2. Csm-mediated synthesis of cA_4 is necessary for activation of the non-sequence specific nuclease Csm6. Related to Figure 1. Detection of *in vitro* transcribed SARS-CoV-2 RNA using the type III surveillance complex $TtCsm^{Csm3-D34A}$ and the ancillary nuclease, TtCsm6. $TtCsm^{Csm3-D34A}$, TtCsm6 and SARS-CoV-2 RNA, but not SARS-CoV-1 RNA, are all required to trigger cleavage of the fluorescent RNA reporter. Addition of cA_4, bypasses the signaling pathway and activates TtCsm6 directly, cleaving the reporter RNA in the absence of SARS-CoV-2 RNA (last lane). These reactions were performed using 10^8 copies of *in vitro* transcribed SARS-CoV-1 or -2 RNA spiked into nasopharyngeal swab clinical matrix. Results from three technical replicates are shown. Results presented in the final column (cA4) demonstrate that the florescent signal is generated by cA_4-dependent activation of Csm6.

Supplementary Fig. 3

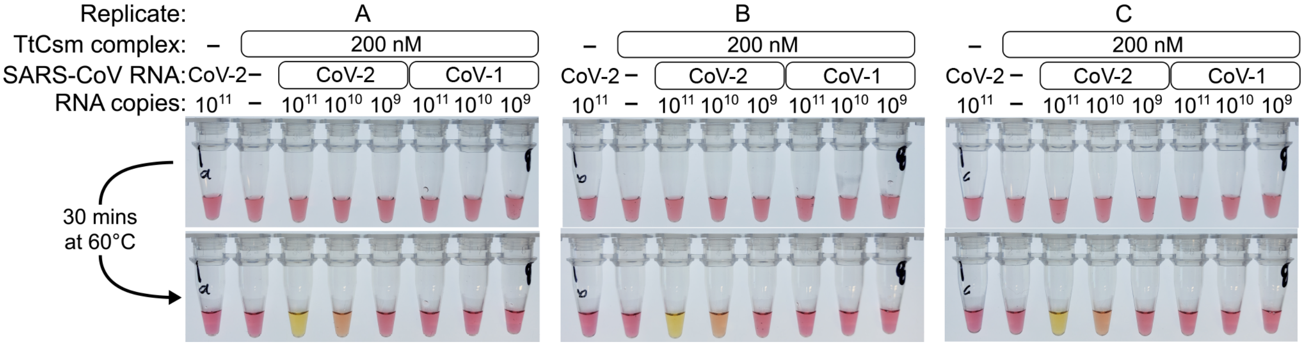

Figure S3. Colorimetric detection of a specific RNA sequence. Related to Figure 1. TtCsm$^{Csm3\text{-}D34A}$ was incubated with either RNA target, in the presence of ATP, for 30 minutes at 60°C. Specific RNA recognition, and associated Cas10-mediated ATP polymerization, causes acidification of the solution, and changes the color of Phenol Red pH indicator from fuchsia, through orange, to yellow. Three technical replicates (A, B and C) are shown.

Supplementary Fig. 4

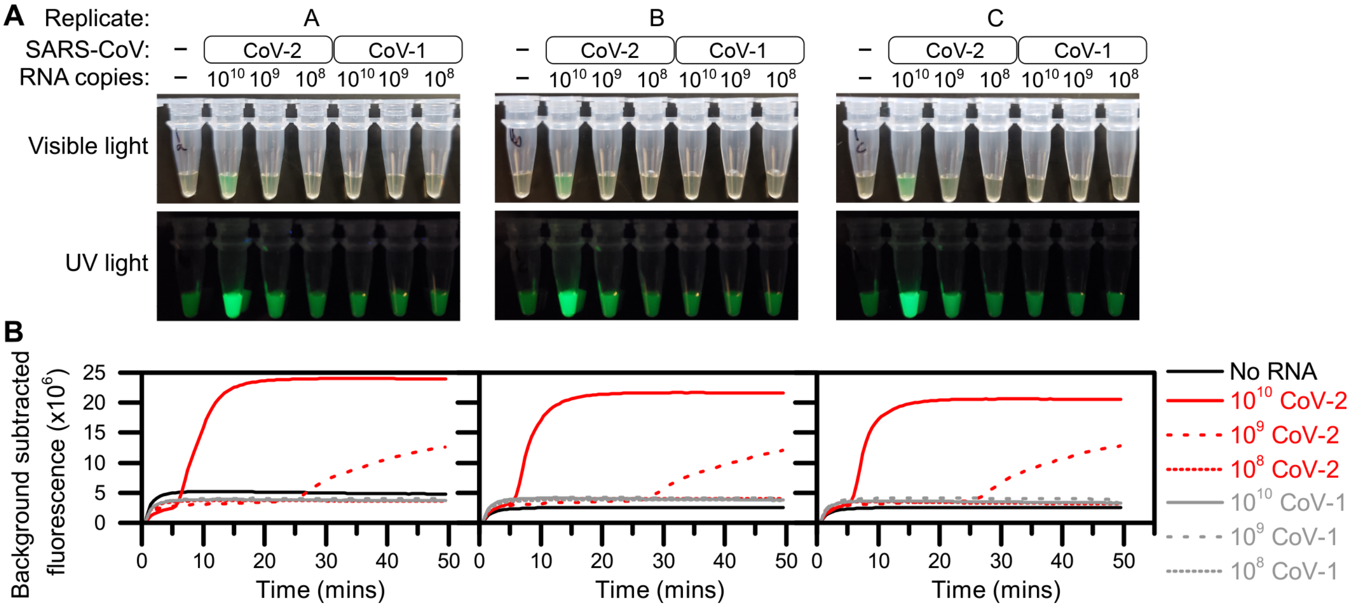

Figure S4. Visible fluorometric detection of a specific RNA sequence. Related to Figure 1. (**A**) TtCsm$^{Csm3\text{-}D34A}$ was incubated with no RNA, a specific (i.e., SARS-CoV-2) or non-specific (SARS-CoV-1) RNA target, in the presence of ATP for 50 minutes at 60°C. The reactions contain an excess of Mg^{2+} relative to Mn^{2+} ions, and the metal indicator Calcein. Calcein preferentially binds Mn^{2+}, forming a quenched complex. Specific RNA recognition, and associated Cas10-mediated ATP polymerization, generates pyrophosphate which precipitates with Mg2+ and Mn2+ ions. Excess Mg^{2+} ions bind Calcein to form a highly fluorescent complex that can be seen by eye in visible light or with UV light. (**B**) Kinetics of Calcein fluorescence for corresponding reactions shown in panel A. 10^{10} SARS-CoV-2 RNAs can be detected in 10 minutes, and 10^9 copies are detected in 40 minutes. No increase in fluorescence is seen beyond the first five minutes for either a no RNA control, or for SARS-CoV-1 RNA containing samples.

Supplementary Fig. 5

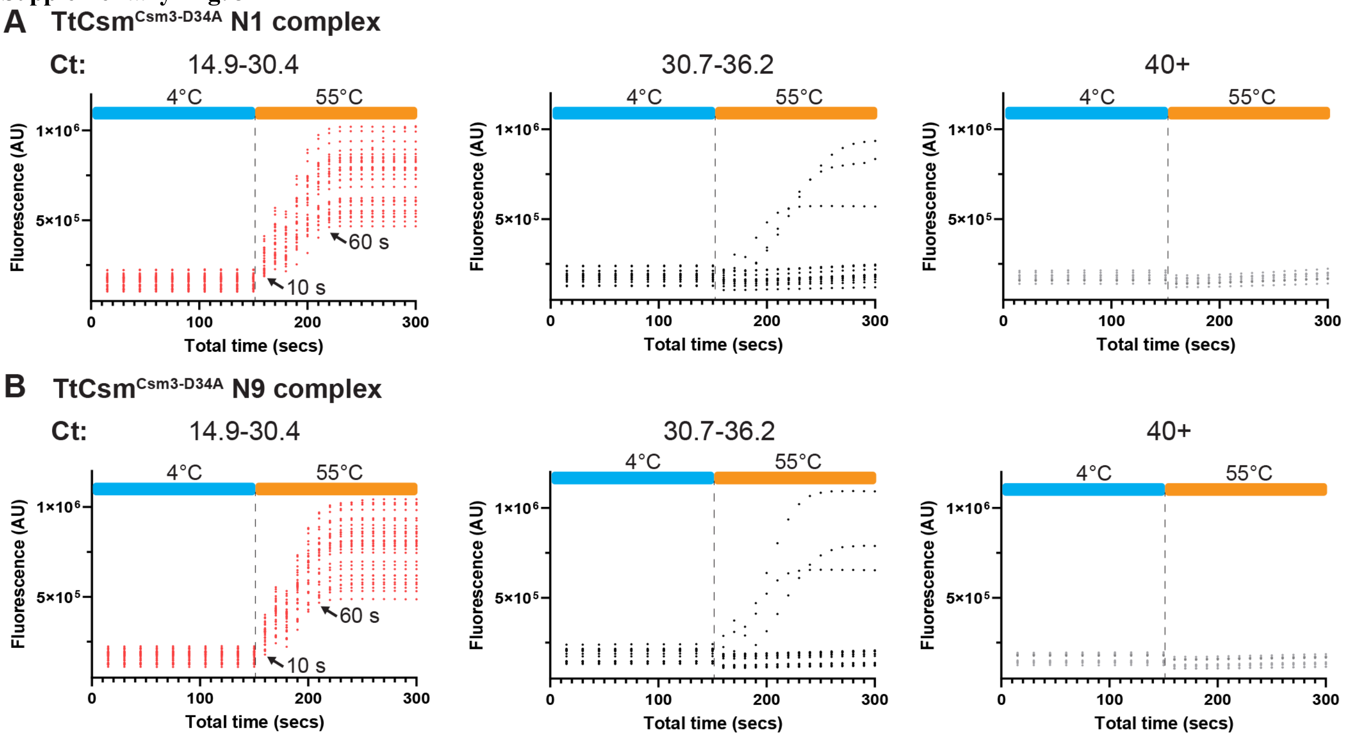

Figure S5. Rapid and specific detection of SARS-CoV-2 by RT-LAMP-T7-Csm. Related to Figure 3. Raw fluorescence kinetics for the T7-Csm stage of RT-LAMP-T7-Csm detection of SARS-CoV-2 RNA from patient samples with Ct values of 14.9-30.4 (left), 30.7-36.2 (middle), and 40+ (right), as detected by the RNase dead TtCsm complex (dTtCsm) loaded with (**A**) crRNA$_{N1}$ or (**B**) crRNA$_{N9}$. Reactions were first incubated in an RT-PCR machine at 4°C and fluorescence was measured every 15 seconds for 150 seconds. Fluorescence readings in both positive and negative reactions are low until the T7-Csm reactions are heated to 55°C, upon which fluorescence rapidly increases within 10 seconds and continues to increase for 60 seconds in most samples.

Supplementary Fig. 6

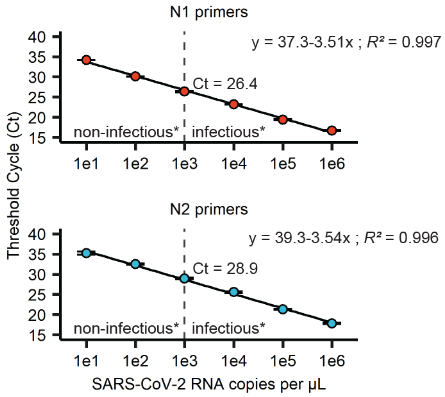

Figure S6. Standard curves for absolute quantification of SARS-CoV-2 titers. Related to Figures 2 and 3. A 10-fold dilution series of SARS-CoV-2 synthetic RNA fragment (RTGM 10169, National Institute of Standards and Technology) containing the Nucleocapsid gene was used. Data was plotted as Cycle Threshold (Ct) on y-axis versus log10 (copies per ml) on x-axis. The mean Ct of three technical replicates are shown, error bars represent ± 1 standard deviation. Trend lines were fit to the data using the geom_smooth function of the ggplot2 R package; linear equations and R2 values are shown. Boundaries corresponding to non-infectious and infectious Ct values refer to observations that patients with viral titers below 10^6 copies/ml are rarely infectious.

Table S1: TtCsm guide RNAs. Related to Figures 1-3 and S1-S5.

Name	***Sequence (5'-3')*** **(target binding region underlined)**
N1 (76 nt guide)	AUUGCGACACGCUGAAGCGCUGGGGGCAAAUUGUGCAAUUUGCGGCCAGUU<br>GCAAGGGAUUGAGCCCCGUAAGGGG
N3 (76 nt guide)	AUUGCGACGGCCGACGUUGUUUUGAUCGCGCCCCACUGCGUUCUCCAUGUUG<br>CAAGGGAUUGAGCCCCGUAAGGGG
N6 (76 nt guide)	AUUGCGACGUUGCGACUACGUGAUGAGGAACGAGAAGAGGCUUGACUGGUU<br>GCAAGGGAUUGAGCCCCGUAAGGGG
N7 (76 nt guide)	AUUGCGACAGCAGCAGCAAAGCAAGAGCAGCAUCACCGCCAUUGCCAGGUU<br>GCAAGGGAUUGAGCCCCGUAAGGGG
N8 (76 nt guide)	AUUGCGACAUGCUUUAGUGGCAGUACGUUUUUGCCGAGGCUUCUUAGAGUU<br>GCAAGGGAUUGAGCCCCGUAAGGGG
N9 (76 nt guide)	AUUGCGACUUCCGAAGAACGCUGAAGCGCUGGGGGCAAAUUGUGCAAUGUU<br>GCAAGGGAUUGAGCCCCGUAAGGGG
N10 (76 nt guide)	AUUGCGACAUUCAGCAAAAUGACUUGAUCUUUGAAAUUUGGAUCUUUGGUU<br>GCAAGGGAUUGAGCCCCGUAAGGGG
N11 (76 nt guide)	AUUGCGACCAGUUUGCUGUUUCUUCUGUCUCUGCGGUAAGGCUUGAGUGUU<br>GCAAGGGAUUGAGCCCCGUAAGGGG
N12 (76 nt guide)	AUUGCGACGUCAGCACUGCUCAUGGAUUGUUGCAAUUGUUUGGAGAAAGUU<br>GCAAGGGAUUGAGCCCCGUAAGGGG
I1 (76 nt guide)	AUUGCGACAAAAGCGAAAACGUUUAUAUAGCCCAUCUGCCUUGUGUGGGUU<br>GCAAGGGAUUGAGCCCCGUAAGGGG

Table S2: Fluorescent reporter RNAs. Related to Figures 1-3 and S5.

Name	*Sequence (5'-3')*	*Associated figure*
RNA reporter A	/56-FAM/rCrUrCrUrCrU/3IABkFQ/	1
RNA reporter B	/56-FAM/rArUrCrUrUrCrUrUrArU/3IABkFQ/	2 and 3

Table S3: Primers to generate IVT RNA templates. Related to Figures 1, 2, S2, S3 and S4

Primer name	***Sequence (5'-3')***
SARS-CoV-2 N1 T7 template Forward	GATAATACGACTCACTATAGGGAACTGATTACAAACATTGGCCGCAAATTGCACAATT
SARS-CoV-2 N1 T7 template Reverse	GCGCGACATTCCGAAGAACGCTGAAGCGCTGGGGGCAAATTGTGCAATTTGCGGCC
SARS-CoV-1 N1 T7 template Forward	GATAATACGACTCACTATAGGGAACTGATTACAAACATTGGCCGCAAATTGCACAATT
SARS-CoV-1 N1 T7 template Reverse	GCGTGACATTCCAAAGAATGCAGAGGCACTTGGAGCAAATTGTGCAATTTGCGGCC
SARS-CoV-2 target all ten complexes_F	CTAGAGCTCGATAATACGACTCACTATAGGGCGTGTTGTTTTAGATTTCATCTAAACG
SARS-CoV-2 target all ten complexes_R	ATCCTGCAGGCACACTGATTAAAGATTGCTATGTG

Table S4: Primers for RT-LAMP. Related to Figures 3 and S5.

Primer name	***Sequence (5'-3')***
F3	GCTGCTGAGGCTTCTAAG
B3	GCGTCAATATGCTTATTCAGC
BIP	TCAGCGTTCTTCGGAATGTCGCTGTGTAGGTCAACCACG
FIP	GCGGCCAATGTTTGTAATCAGTAGACGTGGTCCAGAACAA
Loop Forward	CCTTGTCTGATTAGTTCCTGGT
Loop Reverse	TGGCATGGAAGTCACACC
T7-FIP	TAATACGACTCACTATAGGGAGACGTGGTCCAGAACAA

Table S5: Purified genomic nucleic acids. Related to Figure 3.

Name	***Source***
SARS-CoV-2	The National Institute of Standards and Technology (RGTM 10169)
SARS-CoV-1	American Type Culture Collection (ATCC) (VR-3280SD)
MERS-CoV	ATCC (VR-3248SD)
Human coronavirus HKU1	ATCC (VR-3262SD)
Influenza B	ATCC (VR-1885DQ)
Human coronavirus NL63	ATCC (VR-3263SD)
Human respiratory syncytial virus	ATCC (VR-1580DQ)
Pseudomonas aeruginosa	ATCC (27853D-5)
Candida albicans	ATCC (10231D-5)
