# Supplementary material for: Intrinsic signal amplification by type III CRISPR-Cas systems provides a sequence-specific SARS-CoV-2 diagnostic
Source: Cell Rep Med. 2021 May 27;2(6):100319. doi: 10.1016/j.xcrm.2021.100319 (PMC8157118; doi:10.1016/j.xcrm.2021.100319)
Supplement: Document S2. Article plus supplemental information [file mmc3.pdf]

Cell Reports Medicine

Report

Intrinsic signal amplification by type III CRISPR-Cas systems provides a sequence-specific SARS-CoV-2 diagnostic

Graphical abstract

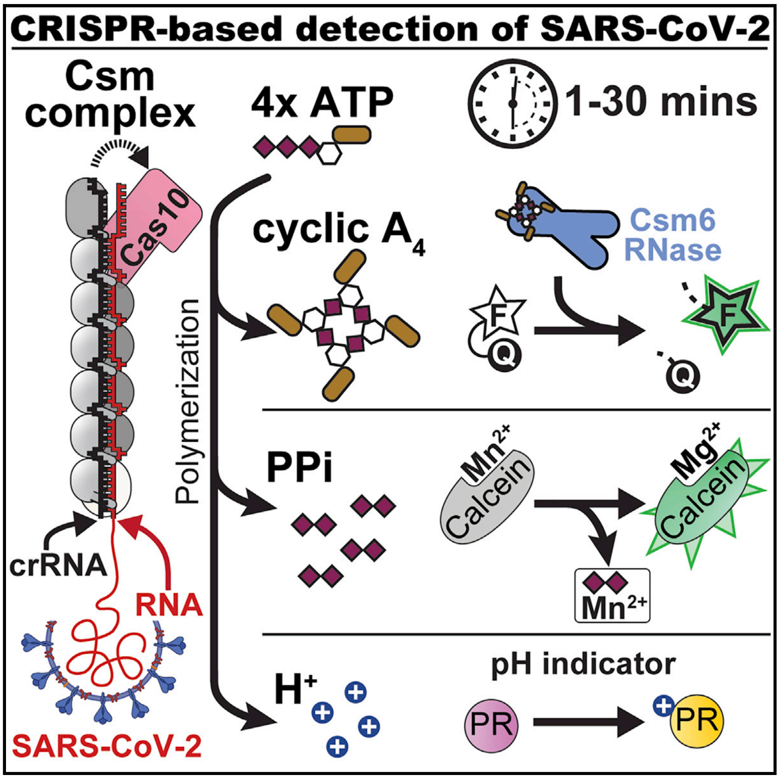

Highlights

- Sequence-specific recognition of RNA by CRISPR Csm complex activates Cas10
- Cas10 polymerizes ATP to make cyclic oligonucleotides, pyrophosphates, and protons
- Cas10's rapidly amplified products are detectable in 1–30 min
- RT-LAMP can be coupled to T7-Csm to rapidly and sensitively detect SARS-CoV-2 RNA

Authors

Andrew Santiago-Frangos, Laina N. Hall, Anna Nemudraia, ..., Mark A. Jutila, Matthew P. Taylor, Blake Wiedenheft

Correspondence

bwiedenheft@gmail.com

In brief

Recognition of a complementary target RNA by the type III CRISPR systems uniquely triggers the activation of a CRISPR-associated polymerase domain in Cas10. The polymerase generates oligoadenylates, protons, and pyrophosphates. Santiago-Frangos et al. repurposed the type III CRISPR-Cas system for sensitive and sequence-specific detection of SARS-CoV-2 by developing three different detection methods that rely on each of these products.

Santiago-Frangos et al., 2021, Cell Reports Medicine 2, 100319
June 15, 2021 © 2021 The Author(s).
https://doi.org/10.1016/j.xcrm.2021.100319

Cell Reports Medicine

CellPress
OPEN ACCESS

Report

Intrinsic signal amplification by type III CRISPR-Cas systems provides a sequence-specific SARS-CoV-2 diagnostic

Andrew Santiago-Frangos,[1,2] Laina N. Hall,[1] Anna Nemudraia,[1] Artem Nemudryi,[1] Pushya Krishna,[1] Tanner Wiegand,[1] Royce A. Wilkinson,[1] Deann T. Snyder,[1] Jodi F. Hedges,[1] Calvin Cicha,[1] Helen H. Lee,[1] Ava Graham,[1] Mark A. Jutila,[1] Matthew P. Taylor,[1] and Blake Wiedenheft[1,3,4,*]

[1]Department of Microbiology and Immunology, Montana State University, Bozeman, MT 59717, USA
[2]Twitter: @SantiagoFrangos
[3]Twitter: @WiedenheftLab
[4]Lead contact
*Correspondence: bwiedenheft@gmail.com
https://doi.org/10.1016/j.xcrm.2021.100319

SUMMARY

There is an urgent need for inexpensive new technologies that enable fast, reliable, and scalable detection of viruses. Here, we repurpose the type III CRISPR-Cas system for sensitive and sequence-specific detection of SARS-CoV-2. RNA recognition by the type III CRISPR complex triggers Cas10-mediated polymerase activity, which simultaneously generates pyrophosphates, protons, and cyclic oligonucleotides. We show that all three Cas10-polymerase products are detectable using colorimetric or fluorometric readouts. We design ten guide RNAs that target conserved regions of SARS-CoV-2 genomes. Multiplexing improves the sensitivity of amplification-free RNA detection from 10^7 copies/μL for a single guide RNA to 10^6 copies/μL for ten guides. To decrease the limit of detection to levels that are clinically relevant, we developed a two-pot reaction consisting of RT-LAMP followed by T7-transcription and type III CRISPR-based detection. The two-pot reaction has a sensitivity of 200 copies/μL and is completed using patient samples in less than 30 min.

INTRODUCTION

Frequent tests and quick results are critical for limiting the spread of severe acute respiratory syndrome-coronavirus-2 (SARS-CoV-2) and ending the current coronavirus disease 2019 (COVID-19) pandemic.[1,2] qRT-PCR (quantitative reverse transcriptase-polymerase chain reaction) has been the gold standard for viral diagnostics, but this method is slow and requires sophisticated equipment that is expensive to purchase and operate. A national survey of individuals tested using nasal swab-based qRT-PCR found that the average wait times for results was 4.1 days, with 10% of tests taking [3]10 days.[3] Thus, there is an urgent need for inexpensive new technologies that enable fast, reliable, and scalable detection of viruses.

Recently, loop-mediated isothermal amplification (LAMP)[4] was developed as a sensitive (1–100 copies/μL) point-of-care diagnostic.[5,6] However, LAMP and reverse-transcription LAMP (RT-LAMP) are prone to generating mis-primed or template-independent amplicons that cause false positive readings if they are not coupled to sequence-specific verification of the amplicon.[7–12] For example, the POP7-targeting primers used in the DETECTR CRISPR-based diagnostic lead to excessive non-specific amplification; however, the sequence-specific verification of the RT-LAMP amplicon by Cas12 yields sensitive and specific detection of POP7 RNA.[12,13] Therefore, platforms that can further verify nucleic acid sequences increase the utility of LAMP and RT-LAMP applications. Type V (Cas12-based) and type VI (Cas13-based) CRISPR systems have been coupled to LAMP or RPA (recombinase polymerase amplification) for sensitive and reliable detection of viral nucleic acids.[13–18] Following isothermal amplification, the RNA-guided Cas12 or Cas13 proteins bind to the amplified target and trigger a non-sequence-specific nuclease activity that cleaves a fluorophore and quencher-labeled DNA or RNA.[14,15,18] Cleavage of the tether results in an increase in fluorescence that can be detected in 45 min. Furthermore, the collateral nuclease activity of Cas13 has previously been used to cleave and mature an RNA oligo into a linear pseudo-ligand for the RNase Csm6, resulting in a 3.5-fold increase signal sensitivity.[14] While Cas12 and Cas13 detection methods have been optimized over several iterations to be compatible with the isothermal amplification of viral RNA, the ultimate goal is to develop CRISPR-based technologies that are sensitive enough to detect the viral RNA directly, without prior amplification. Recently, Fozouni et al.[19] reported that type IV (Cas13a-based) CRISPR systems can be used for amplification-free detection of SARS-CoV-2 RNA in ~30 min and with the sensitivity of ~100 copies/μL.

Like the type VI (Cas13-based) systems, type III systems also target RNA.[20–24] However, type III systems rely on a unique intrinsic signal amplification mechanism (Figure 1). Here, we

© 2021 The Author(s).
This is an open access article under the CC BY license (http://creativecommons.org/licenses/by/4.0/).

set out to determine whether intrinsic amplification by type III systems could be used to detect viral RNA without prior amplification. The proof-of-concept presented here demonstrates that the Csm complex from *Thermus thermophilus* (TtCsm) can be programmed to specifically recognize the SARS-CoV-2 genome. SARS-CoV-2, but not SARS-CoV-1 or a panel of other respiratory pathogens, activates the Cas10 polymerase, which generates ~1,000 cyclic nucleotides (e.g., cA_4) after binding an RNA target.[25–28] Like all polymerases, nucleotide polymerization by Cas10 also generates protons (H^+) and pyrophosphates (PPi). We demonstrate that each of these three products can be used to detect SARS-CoV-2 RNA using either colorimetric, fluorometric, or both methods simultaneously. The assay can be performed in 1–30 min, depending on the detection method and the concentration of the RNA. When coupled with RT-LAMP, the assay can be performed in <30 min and has a limit of detection of ~200 copies/μL. Collectively, this work indicates that the type III systems can be repurposed for fast and sensitive diagnostics.

RESULTS

Sequence-specific activation of Cas10 polymerase yields three detectable products

Sequence-specific recognition of RNA by type III CRISPR systems initiates a signaling cascade (Figure 1A).[26–28] RNA binding by the *Thermus thermophilus* (TtCsm) complex triggers a conformational change that activates the Palm domain of the Cas10 subunit, which amplifies the RNA binding signal by converting ATP into ~1,000 cyclic oligoadenylates (e.g., cA_4).[25–28] We hypothesized that the intrinsic signal amplification unique to type III CRISPR systems would boost the sensitivity of direct RNA detection, while maintaining specificity. To test this hypothesis, we expressed and purified the type III-A CRISPR RNA (crRNA)-guided surveillance complex from TtCsm with a guide complementary to the N-gene of SARS-CoV-2 ($crRNA_{N1}$) (Figures 1B and S1; Table S1).

The Csm3 subunits, which form the "backbone" of the Csm complex, are nucleases that cleave bound target RNA in 6-nt increments.[22,24,29] The cleaved RNA fragments dissociate and the Csm complex returns to the "inactive" state (i.e., no Cas10-polymerase activity) (Figure 1A).[26,30] Thus, in the context of an immune response, the RNase activity of Csm3 moderates Cas10 polymerase activity to limit excess nuclease activation that may otherwise kill the cell.[26,30,31] However, we reasoned that a Csm3 mutation that prevents target RNA degradation would have two related benefits as a diagnostic. First, an RNase-dead Csm complex is expected to stay bound to target RNA longer, which would sustain the Cas10 polymerase activity. Second, Csm3-mediated cleavage of the target RNA (e.g., SARS-CoV-2 RNA) would reduce the target RNA concentration over time and thus limit the sensitivity of the assay. Therefore, we mutated residues in the Csm3 subunit responsible for target RNA cleavage (D34A),[24,29] and purified the RNase-dead complex ($TtCsm^{Csm3\text{-}D34A}$) (Figure S1). To measure the limit of detection (LoD), we added the mutant or wild-type Csm complex to a reaction containing the TtCsm6 nuclease, a fluorescent reporter (i.e., FAM-RNA-Iowa Black FQ), and a titration of RNA corresponding to the N-gene of either SARS-CoV-2 or SARS-CoV-1 (Figures 1C and S2; Tables S2 and S3). Single mismatches in the 3¿ end of target RNAs have been shown to reduce cyclic oligoadenylate production by SepCsm (type III-A) by up to 30-fold as compared to the fully complementary target,[30] while double-nucleotide mutations in the 3′ end of target RNAs ablated cyclic oligoadenylate production by SsoCsm (type III-D).[26] Furthermore, single- and double-nucleotide complementarity between the crRNA 5′ handle and the target 3′ anti-tag significantly decreased the allosteric activation of Cas10 and cyclic oligoadenylate production by SthCsm (type III-A).[32] Single mismatches in the target RNA have been shown to result in 10-fold lower amounts of cyclic oligoadenylate production by other Csm complexes.[30] Using fluorometric detection, both the mutant and the wild-type Csm complex could detect the SARS-CoV-2 RNA at concentrations $>10^8$ copies per reaction, and neither complex cross-reacted with the SARS-CoV-1 RNA at the highest concentrations tested. The RNase-dead TtCsm complex was roughly 3-fold more sensitive than the wild type, with an LoD of ~10^7 copies per reaction.

In addition to fluorometric detection, we developed a colorimetric RNA detection method that uses a pH change that occurs during nucleotide polymerization (Figures 1D and S3). Specific recognition of SARS-CoV-2 by RNase-dead TtCsm complex activates Cas10. Cas10 polymerizes ATP,[25–28] releasing one proton per incorporated nucleotide. Cas10-generated protons acidify the solution and change the color of a pH indicator (i.e., Phenol Red) from fuchsia through orange (10^{10} RNA copies) to yellow (10^{11} RNA copies). Similarly, we developed a visible fluorometric detection method that relies on the sequestration of metallic ions by pyrophosphate. The metal indicator calcein is initially quenched by bound Mn^{2+} ions.[33] In addition to the cyclic oligoadenylates and protons, Cas10 polymerase generates one pyrophosphate per ATP polymerized. Pyrophosphate forms an insoluble precipitate with Mn^{2+}, which unquenches calcein. Free calcein is then bound by excess Mg^{2+}, forming a fluorescent complex that can be seen by eye or with a UV lamp in <10 min (Figures 1E and S4).

Csm-based direct detection of SARS-CoV-2 RNA in patient samples

The LoD using $crRNA_{N1}$ is between 10^7 and 10^8 copies of IVT RNA per microliter, which is insufficient to be clinically relevant (Figures 1C and 2C). To identify other guides that may outperform or complement the activity of $crRNA_{N1}$, we aligned 45,641 SARS-CoV-2 genomes available from GISAID.[34] These alignments were used to select guides based on four key criteria. First, each target sequence had to be >99% identical among the available SARS-CoV-2 genomes. Second, complementarity between the target and the crRNA was not allowed to extend beyond the spacer sequence (guide) and into repeat derived portions of the crRNA that have been shown to suppress Cas10 activity.[27] Third, we targeted regions of SARS-CoV-2 that were different by at least 2 nt in SARS-CoV-1 and MERS-CoV. Fourth, the list of target sequences was pruned to remove guides with similarity to human mRNA sequences or common oral and respiratory pathogen sequences (E value <1,000). Finally, we focused on target sequences located 3¿ of the ORF3a gene, which are

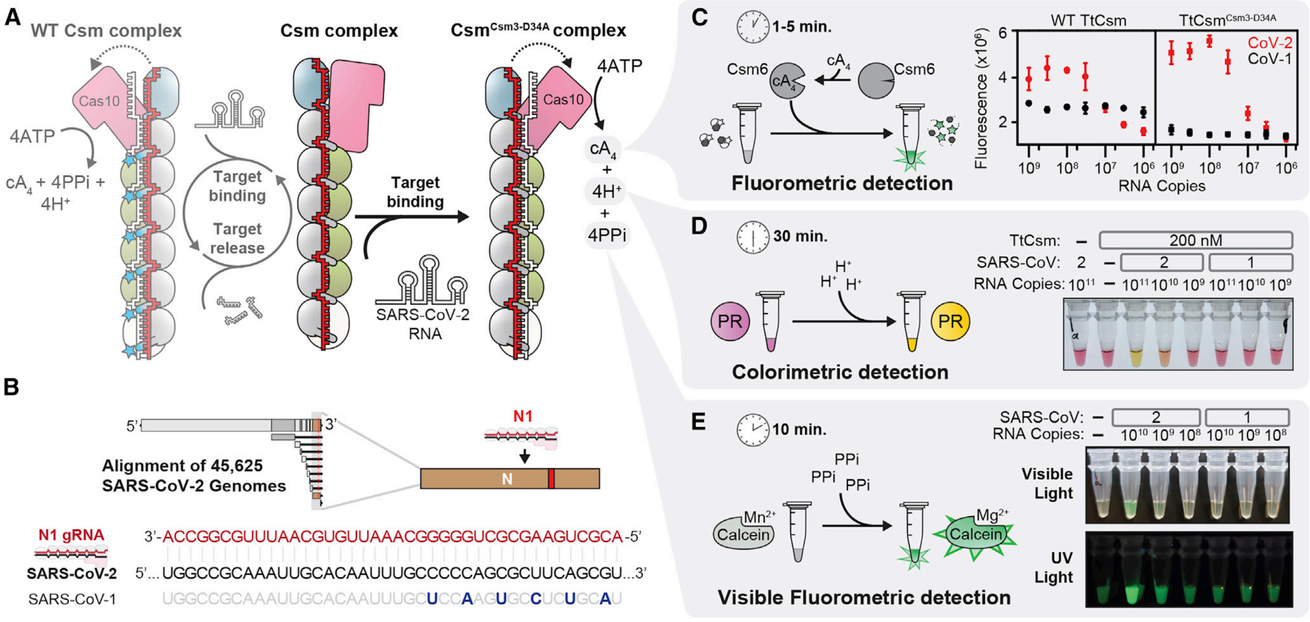

Figure 1. Detection of SARS-CoV-2 using the type III CRISPR-Cas system

(A) Schematic of the type III Csm complex from *Thermus thermophilus* (TtCsm). TtCsm complex consists of a crRNA (red) and an unequal stoichiometry of 5 different proteins ($Cas10_1$, pink: $Csm4_1$, blue: $Csm3_6$, gray: $Csm2_4$, green: $Csm5_1$, white). RNA binding activates the Cas10-polymerase, Cas10 DNase, and Csm3 RNase (left, transparent). Target RNA cleavage by Csm3 subunits (blue stars) causes target dissociation and inactivates Cas10. A mutation in the Csm3 subunit ($TtCsm^{Csm-D34A}$) renders the complex RNase-dead (right).

(B) Schematic of SARS-CoV-2 genome. The N1 CRISPR RNA ($crRNA_{N1}$) targets a sequence on the 3¿ end of genomic and subgenomic RNAs, which is conserved in 45,625 SARS-CoV-2 genomes. Mismatches between the crRNA-guide and SARS-CoV-1 are highlighted (navy blue).

(C) Fluorometric detection of *in vitro* transcribed SARS-CoV-2 (red squares) and SARS-CoV-1 N-gene (black squares). Cyclic tetra-adenylate (cA_4) activates TtCsm6, which is a non-sequence-specific ancillary nuclease (Figure S2). Activated TtCsm6 cleaves an RNA tether, which links a fluorophore (star) to a quencher (gray hexagon). $TtCsm^{Csm3-D34A}$ N1 (right graph) exhibits a LoD 3-fold lower than wild-type TtCsm N1 (left graph) and retains specificity for SARS-CoV-2 RNA. The mean of 3 technical replicates are shown; error bars represent ±1 SD.

(D) Colorimetric detection of SARS-CoV-2 RNA by $TtCsm^{Csm3-D34A}$ N1 complex using a pH-sensitive dye (Phenol Red). Reactions were incubated for 30 min at 60°C. Technical replicates are shown in Figure S3.

(E) Visible fluorometric detection of SARS-CoV-2 RNA by $TtCsm^{Csm3-D34A}$ N1 complex using calcein. Reactions were incubated for up to 1 h at 60°C. Technical replicates and kinetics are shown in Figure S4.

present on both the viral genome and subgenomic RNAs generated during infection. In total, we designed crRNAs targeting 10 different locations on the SARS-CoV-2 genome (Figures 2A and S1; Table S1). To determine how each of these guides perform, we measured sequence-specific detection of RNA using a fluorometric reporter assay (i.e., FAM-RNA-Iowa Black FQ) (Figure 2B). Most of the crRNAs provide similar sensitivity; however, $crRNA_{N1}$ and $crRNA_{N9}$ generated significantly more signal than the next best complex tested ($p < 0.0001$). We then tested $crRNA_{N1}$ and $crRNA_{N9}$ on RNA isolated from the nasal swab of an infected patient (Figure 2C). Both $crRNA_{N1}$- and $crRNA_{N9}$-guided complexes generate a similar signal for either IVT RNA or RNA isolated from a SARS-CoV-2$^+$ patient (i.e., 2- to 3-fold increase in signal by 5 min, relative to the first time point). The LoD for $crRNA_{N1}$ and $crRNA_{N9}$ is 10^7 copies/μL (Figure 2D) ($p < 0.0001$).

Fozouni et al.[19] recently showed that multiplexing Cas13 (i.e., combining multiple guides into a single reaction) improves the sensitivity of SARS CoV-2 detection. We reasoned that similar benefits may be possible for Csm-based detection. To test this idea, we combined 10 of the guides (2.5 nM each) into a single multiplexed reaction. Multiplexing 10 guides improves the sensitivity of TtCsm-mediated detection of SARS-CoV-2 RNA isolated from the nasal swab of a positive patient by ~10 times (Figures 2C and 2D). However, the sensitivity of direct detection appears to increase additively with the number of TtCsm complexes, which precludesthe direct detection of RNA at concentrations that are clinically relevant.

Testing clinical samples for SARS-CoV-2 using RT-LAMP and T7-Csm

Csm-based detection is currently not sensitive enough to directly detect SARS-CoV-2 in all of the patients capable of spreading the infection, which requires an LoD of 10^3 RNA copies/μL[1,2,35,36] (Figure 2C). To decrease the LoD of a type III CRISPR-based diagnostic to £10^3 RNA copies/μL, we incorporated an upstream nucleic acid amplification technique (Figure 3A). SARS-CoV-2 genomic RNA is reverse transcribed (RT) into DNA, which is then amplified by LAMP using primers that flank regions of the SARS-CoV-2 genome targeted by $crRNA_{N1}$ and $crRNA_{N9}$. One of the LAMP primers incorporates a T7 promoter into the amplified DNA, which is then used for *in vitro*

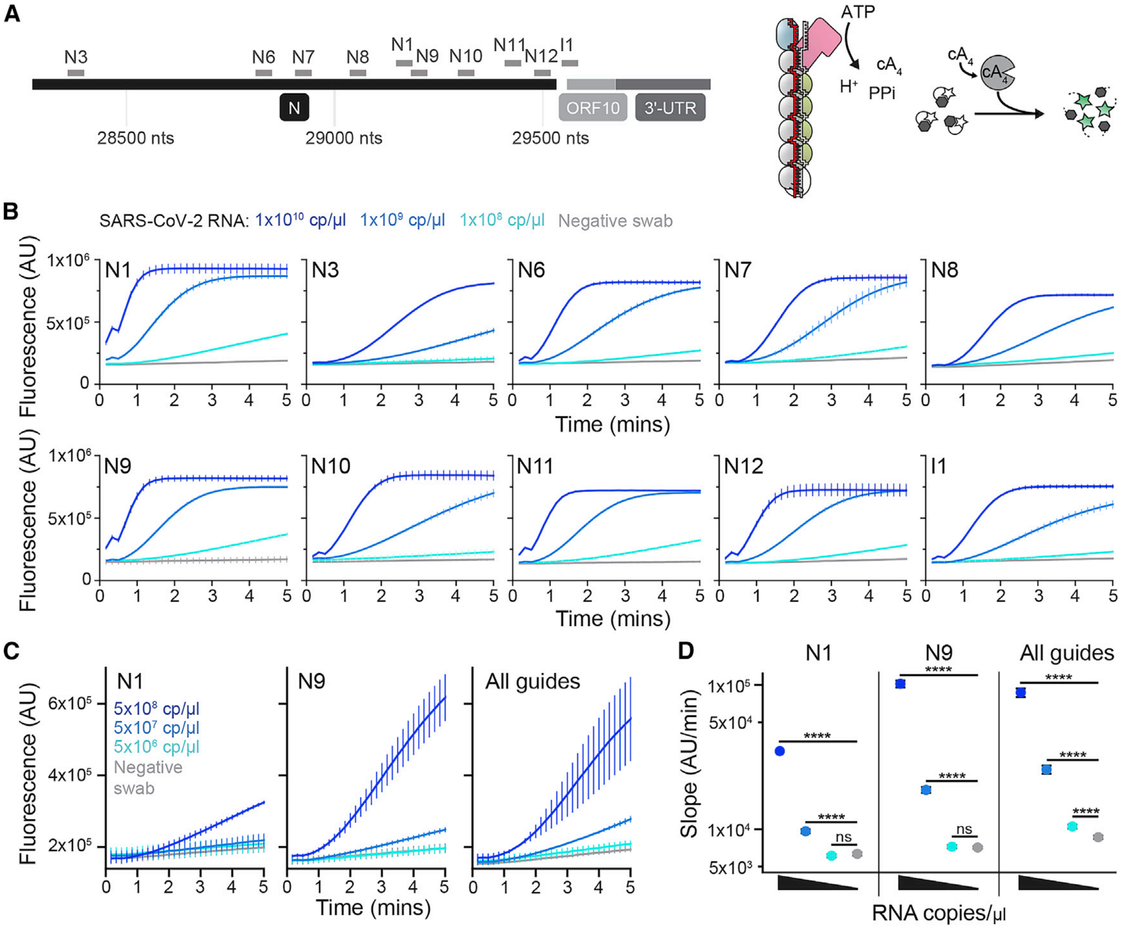

Figure 2. Screening and multiplexing crRNA guides

(A) Regions of SARS-CoV-2 genome targeted by each of the 10 guides, and schematic of RNA reporter-based assay (right) used to test guides.

(B) Detection of SARS-CoV-2 IVT RNA, spiked into RNA extracted from patients lacking a SARS-CoV-2 infection, by 10 different TtCsm$^{Csm3-D34A}$ complexes (25 nM), via a reporter RNA-based assay (Figure 1C; Tables S1 and S3). Means and SDs of 2 technical replicates are shown.

(C) Direct detection of SARS-CoV-2 genomes in RNA extracted from patient samples by 25 nM TtCsm$^{Csm3-D34A}$ N1 or N9, or a mixture of 10 different TtCsm$^{Csm3-D34A}$ complexes, each at 2.5 nM, via a reporter RNA-based assay. RNA extracted from a patient with a high viral load (5×10^8 copies/µL as determined by qRT-PCR) was diluted into RNA extracted from patients lacking a SARS-CoV-2 infection. Means and SDs of 3 technical replicates are shown.

(D) Slopes of increasing fluorescence, measured in (C), were calculated by simple linear regression. The calculated slope and ±95% confidence intervals are shown. Positive RNA slopes were compared to the negative swab RNA slope by an F-test: ****$p < 0.0001$, ns, not significantly higher than negative swab RNA control.

transcription (T7) and detected by TtCsm (Figures 3A and S5; Table S4).

To confirm the specificity of TtCsm-based detection, we tested SARS-CoV-2 alongside a panel of eight other oral and respiratory pathogens, including coronaviruses SARS-CoV-1, Middle East respiratory syndrome coronavirus (MERS-CoV), human coronavirus HKU1, and human coronavirus NL63 (Figure 3B). These samples resulted in a background signal similar to the no-template control (NTC) (Figure 3B). In contrast, SARS-CoV-2 RNA results in a 4- to 5-fold increase in signal.

To determine the LoD of RT-LAMP-T7-Csm, we tested 20 replicates of 2-fold serial dilutions ranging from ~100-400 copies/µL SARS-CoV-2 RNA (Figure 3C). The LoD of RT-LAMP-T7-Csm is 198 copies/µL SARS-CoV-2 RNA (20/20 replicates), in an assay that relies on a 29-min RT-LAMP step, followed by a 1-min T7-Csm fluorometric detection reaction (Figure 3D; Table 1).

To further validate this method, we next tested RNA extracted from 56 nasopharyngeal swab samples taken from patients who had previously been tested using qRT-PCR. Of the 56 samples tested, 46 were positive for SARS-CoV-2 and 10 were negative by qRT-PCR (Figure 3E). Using two different crRNA guides, we demonstrate that the type III CRISPR system has a specificity (negative predictive agreement) of 100%, as well as a positive predictive agreement of 100% for nasopharyngeal swab samples with 100–200 copies/µL SARS-CoV-2 RNA as determined by qRT-PCR (Figures 3E and S5). Whole-genome sequencing revealed that three of the patient samples used here belonged to the B.1.1.7. lineage. These genome sequences have been deposited in GISAID

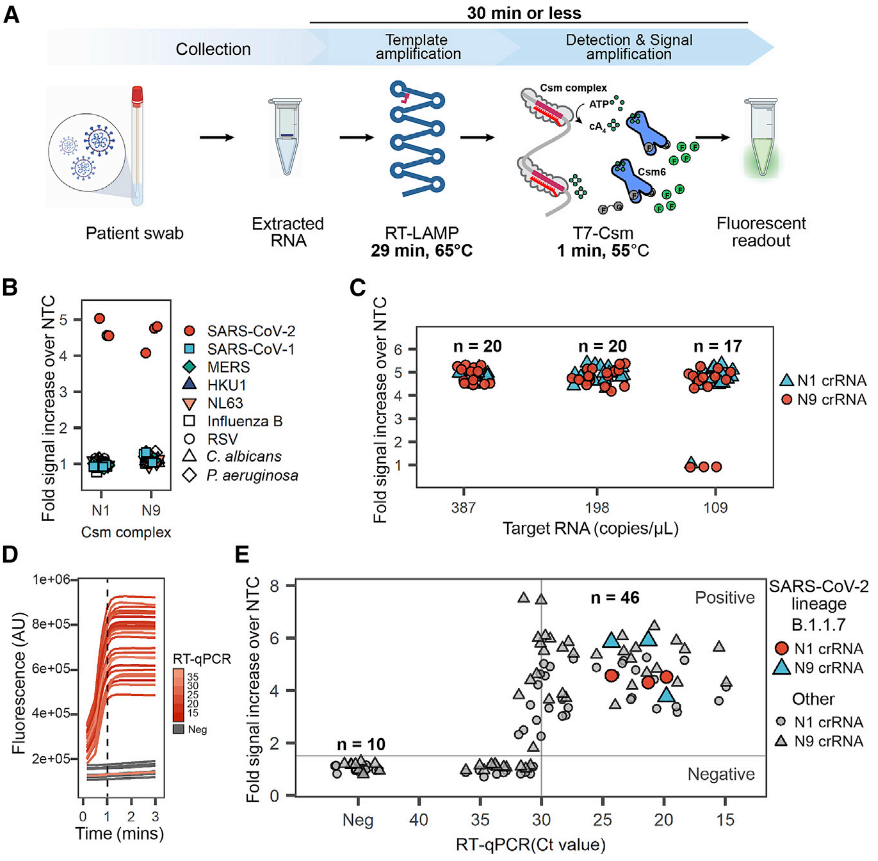

Figure 3. CRISPR-Csm-based detection of SARS-CoV-2 in clinical nasopharyngeal swab samples

(A) Schematic of RT-LAMP-T7-Csm based detection. The viral RNA is reverse transcribed, and the resulting DNA is amplified in an RT-LAMP reaction to produce transcription templates for T7 RNA polymerase, in one pot. An aliquot of the RT-LAMP reaction (29 min) is then mixed with the T7-Csm reaction (1 min).

(B) RT-LAMP-T7-Csm is specific. Neither $crRNA_{N1}$ or $crRNA_{N9}$ cross-react with other coronaviruses, or other common human oral pathogens or flora. Detection of SARS-CoV-2 by both crRNAs is rapid, 1 min, and robust, 4- to 5-fold increase in signal over no-template control (NTC). Three technical replicates are shown.

(C) RT-LAMP-T7-Csm is sensitive. $TtCsm^{Csm3\text{-}D34A}$ complexes loaded with either $crRNA_{N1}$ or $crRNA_{N9}$ have LoDs of 198 copies/μL (20/20 technical replicates); 20 technical replicates are shown.

(D) Kinetics of fluorescence signal increase in T7-Csm reactions. SARS-CoV-2^{+} patient samples are observed to have a ~2-fold increase in signal over NTC by 10 s ($crRNA_{N1}$ median = 1.8, $crRNA_{N9}$ median = 2.3), which rapidly increases to a 4-5-fold increase in signal over an NTC reaction by 1 min ($crRNA_{N1}$ median = 4.2, $crRNA_{N9}$ median = 5.5). A subset of traces is shown for clarity (Figure S5).

(E) Nasopharyngeal swabs from 56 individuals were tested with qRT-PCR (x axis) and RT-LAMP-T7-Csm (y axis). Swabs with Ct values <40 for both N1 and N2 CDC diagnostic primers are considered positive for SARS-CoV-2 RNA. RT-LAMP-T7-Csm reliably identifies patient samples with a Ct < 30.7 (200–100 RNA copies/μL) as positive for SARS-CoV-2 (Figure S6). The B.1.1.7 variant is positively identified by both $crRNA_{N1}$ and $crRNA_{N9}$. Data are shown as fold change in fluorescence as compared to NTC reaction. Single technical replicates are shown for qRT-PCR and RT-LAMP-T7-Csm experiments with either N1 or N9 crRNAs.

(accession IDs: EPI_ISL_1081321, EPI-ISL_1081322, EPI_ISL_1081323).[34] Importantly, the B.1.1.7. variants were positively identified by RT-LAMP-T7-Csm with both N1 and N9 crRNA guides (Figure 3E; N1, red circles; N9, blue triangles).

DISCUSSION

Collectively, this work demonstrates that sequence-specific detection of viral RNA by the type III CRISPR-Cas complex triggers the synthesis of cA_4, pyrophosphate, and protons, each of which are detectable within 1–30 min, using colorimetric or fluorometric methods (Figure 1). Coupling RT-LAMP to T7 transcription and Csm-based detection creates a rapid (<30 min) testing protocol with attomolar sensitivity and high specificity. Moreover, we show that this protocol is capable of detecting the B.1.1.7 SARS-CoV-2 variant, and we anticipate that the guide design criteria described here could be applied to the specific detection of other pathogens. Furthermore, the RT-LAMP and T7-Csm reactions occur in similar buffers and at similar temperatures; therefore, it is possible that additional modifications that either increase the efficiency of RT-LAMP at 55°C (i.e., redesigning primer sets), or that increase the efficiency of T7-Csm at 65°C (i.e., a more thermostable RNA polymerase), could enable an integrated one-pot diagnostic.

While the LoD for RT-LAMP-T7-Csm is 1–1.5 orders of magnitude higher than FDA Emergency Use Authorization (EUA)-approved DETECTR and SHERLOCK assays (20 and 6.75 copies/μL, respectively), the time to result is ~30% faster, which would translate to a higher throughput of samples analyzed per unit of machine run time. Sensitivity was strategically sacrificed for time in this proof-of-concept application for optimal detection of infectious SARS-CoV-2 patients, which requires an LoD of 1,000 copies/μL.[1,2,35,36] However, an increase in the length of the RT-LAMP step (i.e., to 40 min, as is used in SHERLOCK), would likely increase the sensitivity. Additional modifications that could further increase sensitivity include the optimization of RT-LAMP primer sets, a screen for more active type III Csm or Cmr surveillance complexes, or a screen for CARF-fusion effectors that do not cleave their cyclic oligoadenylate ligands.[39] Alternatively, more sensitive methods of measuring Cas10-generated protons, such as ion-sensitive field-effector transistors used in label-free sequencing by synthesis technologies, could decrease the LoD.

Type V (Cas12-based) and type VI (Cas13-based) CRISPR-based diagnostics hinge completely on their collateral nuclease

Table 1. Comparison of clinical parameters of RT-LAMP-T7-Csm and FDA EUA-approved protocols of DETECTR and SHERLOCK diagnostics[37,38]

Test	Time to result (min)	LoD (copies/μL)	Positive agreement (%)		Negative agreement (%)
			^{3}2x LoD	All samples tested	All samples tested
DETECTR	45	20	95	95	100
SHERLOCK	50	6.75	100	100	100
RT-LAMP-T7-Csm	30	200	100	74	100

EUA, Emergency Use Authorization; FDA, US Food and Drug Administration; LoD, limit of detection.

activity.[13–17,40] The Cas10 subunits of Csm complexes possess an analogous nuclease activity triggered upon target binding.[21,29,41–43] However, here, we focused on the NTP polymerase activity of the activated Csm complex, which enables readouts based on several different chemistries (Figure 1). Furthermore, there is a rich resource of naturally occurring downstream effector proteins that have evolved to be activated by the Cas10 polymerized NTP products, which possess a wide range of enzymatic activities. These effectors include RNases, DNases, putative proteases, nitrilases, adenosine deaminases, and adenylate cyclases.[44] Future efforts are aimed at incorporating complementary effectors with the goal of reducing the LoD for the direct detection of RNA in clinical samples.

Limitations of the study

This study demonstrates a proof-of-concept for a sensitive, specific, and rapid diagnostic based on the type III CRISPR system. The current implementation requires pre-amplification using RT-LAMP, followed by T7-transcription and type III detection (T3D). Clinical implementation of this approach will benefit from changes that eliminate the need for RT-LAMP and T7 amplification, improvements that limit sample handling, as well as a better understanding of the base pairing requirements that activate the Cas10 polymerase.[16,19,26,30,32] Improvements will benefit from screening additional guides, testing other type III complexes for accelerated polymerase activity, and incorporating other ancillary nucleases or other effectors in a way that boosts the sensitivity and reduces the time to result.

STAR★METHODS

Detailed methods are provided in the online version of this paper and include the following:

- KEY RESOURCES TABLE
- RESOURCE AVAILABILITY
  - ○ Lead contact
  - ○ Materials availability
  - ○ Data and code availability
- EXPERIMENTAL MODEL AND SUBJECT DETAILS
- METHOD DETAILS
  - ○ Nucleic acid preparation
  - ○ Plasmids
  - ○ Protein purifications
  - ○ Type III CRISPR-based RNA detection
  - ○ Colorimetric CRISPR-Csm based detection
  - ○ Visible fluorometric CRISPR-Csm based detection
  - ○ RT-LAMP-T7-Csm
  - ○ Human clinical sample collection and preparation
  - ○ RT-qPCR
  - ○ Bioinformatic design of TtCsm crRNA guides targeting SARS-CoV-2
  - ○ Sequencing of SARS-CoV-2 RNA isolated from patient samples
- QUANTIFICATION AND STATISTICAL ANALYSIS

SUPPLEMENTAL INFORMATION

Supplemental information can be found online at https://doi.org/10.1016/j.xcrm.2021.100319.

ACKNOWLEDGMENTS

We are grateful to members of Bozeman Health who provided deidentified patient samples, specifically Christopher Nero, Douglas Smoot, Winfield Wallace, Cayley Faurot-Daniels, Verena Lawrence, and Melissa Blauvelt. We are also grateful to Michelle Flenniken, Katie Daughenbaugh, Diane Bimczok, and other members of the COVID task force at Montana State University (MSU) for assistance establishing the COVID testing center. A.S.-F. is a postdoctoral fellow of the Life Science Research Foundation, which is supported by the Simons Foundation. A.S.-F. is supported by the Postdoctoral Enrichment Program Award from the Burroughs Wellcome Fund. Research in the Wiedenheft lab and the biosafety level 3 facility is supported by the Office of the Vice President for Research at Montana State University, a generous donation from the Rosolowsky family, and a sponsored research agreement from VIRIS Detection Systems. Some graphical assets were created with BioRender.com.

AUTHOR CONTRIBUTIONS

B.W., A.S.-F., A. Nemudraia, and A. Nemudryi conceived the experimental plans. A.S.-F. and R.A.W. conducted the fluorometric and colorimetric Csm-based RNA detection. A.S.-F. conducted the visual fluorometric Csm-based RNA detection. L.N.H., P.K., A.G., and R.A.W. purified the proteins. A. Nemudraia and A. Nemudryi performed the qRT-PCR. A.S.-F., A. Nemudraia, and A. Nemudryi performed the RT-LAMP-T7-Csm-based RNA detection. T.W. performed the bioinformatic analyses and designed the crRNA guides. C.C. performed sequencing and analysis of the SARS-CoV-2 genomes. D.T.S., J.F.H., and H.H.L. performed the RNA extraction of patient nasopharyngeal swab samples under the guidance of M.A.J. and M.P.T. A.S.-F., T.W., A. Nemudraia, A. Nemudryi, and B.W. wrote the manuscript.

DECLARATION OF INTERESTS

B.W. is the founder of SurGene and VIRIS Detection Systems. B.W., A.S.-F., A. Nemudraia, and A. Nemudryi are inventors on patent applications related to CRISPR-Cas systems and applications thereof.

INCLUSION AND DIVERSITY

One or more of the authors of this paper self-identifies as an underrepresented ethnic minority in science. One or more of the authors of this paper self-identifies as a member of the LGBTQ+ community. One or more of the authors of this paper received support from a program designed to increase minority representation in science. While citing references scientifically relevant for this work, we also actively worked to promote gender balance in our reference list.

Received: October 8, 2020
Revised: March 4, 2021
Accepted: May 20, 2021
Published: May 27, 2021

REFERENCES

1. Larremore, D.B., Wilder, B., Lester, E., Shehata, S., Burke, J.M., Hay, J.A., Tambe, M., Mina, M.J., and Parker, R. (2021). Test sensitivity is secondary to frequency and turnaround time for COVID-19 screening. Sci. Adv. *7*, 1–11.
2. Paltiel, A.D., Zheng, A., and Walensky, R.P. (2020). Assessment of SARS-CoV-2 Screening Strategies to Permit the Safe Reopening of College Campuses in the United States. JAMA Netw. Open *3*, e2016818.
3. Lazer, D., Santillana, M., Perlis, R.H., Ognyanova, K., Baum, M.A., Quintana, A., Druckman, J., Volpe, J.D., Chwe, H., and Simonson, M. (2020). State of the Nation: A 50-State COVID-19 Survey: Report #8: Failing the Test: Waiting Times for COVID Diagnostic Tests Across the U.S. https://search.bvsalud.org/global-literature-on-novel-coronavirus-2019-ncov/resource/en/grc-740424.
4. Notomi, T., Okayama, H., Masubuchi, H., Yonekawa, T., Watanabe, K., Amino, N., and Hase, T. (2000). Loop-mediated isothermal amplification of DNA. Nucleic Acids Res. *28*, E63.
5. Dao Thi, V.L., Herbst, K., Boerner, K., Meurer, M., Kremer, L.P., Kirrmaier, D., Freistaedter, A., Papagiannidis, D., Galmozzi, C., Stanifer, M.L., et al. (2020). A colorimetric RT-LAMP assay and LAMP-sequencing for detecting SARS-CoV-2 RNA in clinical samples. Sci. Transl. Med. *12*, 1–20.
6. Zhang, Y., Ren, G., Buss, J., Barry, A.J., Patton, G.C., and Tanner, N.A. (2020). Enhancing colorimetric loop-mediated isothermal amplification speed and sensitivity with guanidine chloride. Biotechniques *69*, 178–185.
7. Meagher, R.J., Priye, A., Light, Y.K., Huang, C., and Wang, E. (2018). Impact of primer dimers and self-amplifying hairpins on reverse transcription loop-mediated isothermal amplification detection of viral RNA. Analyst (Lond.) *143*, 1924–1933.
8. Hardinge, P., and Murray, J.A.H. (2019). Reduced False Positives and Improved Reporting of Loop-Mediated Isothermal Amplification using Quenched Fluorescent Primers. Sci. Rep. *9*, 7400.
9. Gao, X., Sun, B., and Guan, Y. (2019). Pullulan reduces the non-specific amplification of loop-mediated isothermal amplification (LAMP). Anal. Bioanal. Chem. *411*, 1211–1218.
10. Rolando, J.C., Jue, E., Barlow, J.T., and Ismagilov, R.F. (2020). Real-time kinetics and high-resolution melt curves in single-molecule digital LAMP to differentiate and study specific and non-specific amplification. Nucleic Acids Res. *48*, e42.
11. Zou, Y., Mason, M.G., and Botella, J.R. (2020). Evaluation and improvement of isothermal amplification methods for point-of-need plant disease diagnostics. PLoS One *15*, e0235216.
12. Ooi, K.H., Liu, M.M., Tay, J.W.D., Teo, S.Y., Kaewsapsak, P., Jin, S., Lee, C.K., Hou, J., Maurer-Stroh, S., Lin, W., et al. (2021). An engineered CRISPR-Cas12a variant and DNA-RNA hybrid guides enable robust and rapid COVID-19 testing. Nat. Commun. *12*, 1739.
13. Broughton, J.P., Deng, X., Yu, G., Fasching, C.L., Servellita, V., Singh, J., Miao, X., Streithorst, J.A., Granados, A., Sotomayor-Gonzalez, A., et al. (2020). CRISPR–Cas12-based detection of SARS-CoV-2. Nat. Biotechnol. *38*, 870–874.
14. Gootenberg, J.S., Abudayyeh, O.O., Kellner, M.J., Joung, J., Collins, J.J., and Zhang, F. (2018). Multiplexed and portable nucleic acid detection platform with Cas13, Cas12a and Csm6. Science *360*, 439–444.
15. Chen, J.S., Ma, E., Harrington, L.B., Da Costa, M., Tian, X., Palefsky, J.M., and Doudna, J.A. (2018). CRISPR-Cas12a target binding unleashes indiscriminate single-stranded DNase activity. Science *360*, 436–439.
16. Joung, J., Ladha, A., Saito, M., Kim, N.-G., Woolley, A.E., Segel, M., Barretto, R.P.J., Ranu, A., Macrae, R.K., Faure, G., et al. (2020). Detection of SARS-CoV-2 with SHERLOCK One-Pot Testing. N. Engl. J. Med. *383*, 1492–1494.
17. Gootenberg, J.S., Abudayyeh, O.O., Lee, J.W., Essletzbichler, P., Dy, A.J., Joung, J., Verdine, V., Donghia, N., Daringer, N.M., Freije, C.A., et al. (2017). Nucleic acid detection with CRISPR-Cas13a/C2c2. Science *356*, 438–442.
18. East-Seletsky, A., O'Connell, M.R., Knight, S.C., Burstein, D., Cate, J.H.D., Tjian, R., and Doudna, J.A. (2016). Two distinct RNase activities of CRISPR-C2c2 enable guide-RNA processing and RNA detection. Nature *538*, 270–273.
19. Fozouni, P., Son, S., Díaz de León Derby, M., Knott, G.J., Gray, C.N., D'Ambrosio, M.V., Zhao, C., Switz, N.A., Kumar, G.R., Stephens, S.I., et al. (2021). Amplification-free detection of SARS-CoV-2 with CRISPR-Cas13a and mobile phone microscopy. Cell *184*, 323–333.e9.
20. Hale, C.R., Zhao, P., Olson, S., Duff, M.O., Graveley, B.R., Wells, L., Terns, R.M., and Terns, M.P. (2009). RNA-guided RNA cleavage by a CRISPR RNA-Cas protein complex. Cell *139*, 945–956.
21. Kazlauskiene, M., Tamulaitis, G., Kostiuk, G., Venclovas, Č., and Siksnys, V. (2016). Spatiotemporal Control of Type III-A CRISPR-Cas Immunity: Coupling DNA Degradation with the Target RNA Recognition. Mol. Cell *62*, 295–306.
22. Samai, P., Pyenson, N., Jiang, W., Goldberg, G.W., Hatoum-Aslan, A., and Marraffini, L.A. (2015). Co-transcriptional DNA and RNA cleavage during type III CRISPR-cas immunity. Cell *161*, 1164–1174.
23. Staals, R.H.J., Zhu, Y., Taylor, D.W., Kornfeld, J.E., Sharma, K., Barendregt, A., Koehorst, J.J., Vlot, M., Neupane, N., Varossieau, K., et al. (2014). RNA targeting by the type III-A CRISPR-Cas Csm complex of Thermus thermophilus. Mol. Cell *56*, 518–530.
24. Tamulaitis, G., Kazlauskiene, M., Manakova, E., Venclovas, Č., Nwokeoji, A.O., Dickman, M.J., Horvath, P., and Siksnys, V. (2014). Programmable RNA shredding by the type III-A CRISPR-Cas system of Streptococcus thermophilus. Mol. Cell *56*, 506–517.
25. Jia, N., Jones, R., Sukenick, G., and Patel, D.J. (2019). Second Messenger cA_4 Formation within the Composite Csm1 Palm Pocket of Type III-A CRISPR-Cas Csm Complex and Its Release Path. Mol. Cell *75*, 933–943.e6.
26. Rouillon, C., Athukoralage, J.S., Graham, S., Grüschow, S., and White, M.F. (2018). Control of cyclic oligoadenylate synthesis in a type III CRISPR system. eLife *7*, e36734.
27. Kazlauskiene, M., Kostiuk, G., Venclovas, Č., Tamulaitis, G., and Siksnys, V. (2017). A cyclic oligonucleotide signaling pathway in type III CRISPR-Cas systems. Science *357*, 605–609.
28. Niewoehner, O., Garcia-Doval, C., Rostøl, J.T., Berk, C., Schwede, F., Bigler, L., Hall, J., Marraffini, L.A., and Jinek, M. (2017). Type III CRISPR-Cas systems produce cyclic oligoadenylate second messengers. Nature *548*, 543–548.
29. Liu, T.Y., Iavarone, A.T., and Doudna, J.A. (2017). RNA and DNA targeting by a reconstituted Thermus thermophiles Type III-A CRISPR-Cas system. PLoS ONE *12*, e0170552.
30. Nasef, M., Muffly, M.C., Beckman, A.B., Rowe, S.J., Walker, F.C., Hatoum-Aslan, A., and Dunkle, J.A. (2019). Regulation of cyclic oligoadenylate synthesis by the *Staphylococcus epidermidis* Cas10-Csm complex. RNA *25*, 948–962.
31. Athukoralage, J.S., Graham, S., Rouillon, C., Grüschow, S., Czekster, C.M., and White, M.F. (2020). The dynamic interplay of host and viral enzymes in type III CRISPR-mediated cyclic nucleotide signalling. eLife *9*, e55852.
32. You, L., Ma, J., Wang, J., Artamonova, D., Wang, M., Liu, L., Xiang, H., Severinov, K., Zhang, X., and Wang, Y. (2019). Structure Studies of the CRISPR-Csm Complex Reveal Mechanism of Co-transcriptional Interference. Cell *176*, 239–253.e16.

33. Tomita, N., Mori, Y., Kanda, H., and Notomi, T. (2008). Loop-mediated isothermal amplification (LAMP) of gene sequences and simple visual detection of products. Nat. Protoc. *3*, 877–882.

34. Elbe, S., and Buckland-Merrett, G. (2017). Data, disease and diplomacy: GISAID's innovative contribution to global health. Glob. Chall. *1*, 33–46.

35. La Scola, B., Le Bideau, M., Andreani, J., Hoang, V.T., Grimaldier, C., Colson, P., Gautret, P., and Raoult, D. (2020). Viral RNA load as determined by cell culture as a management tool for discharge of SARS-CoV-2 patients from infectious disease wards. Eur. J. Clin. Microbiol. Infect. Dis. *39*, 1059–1061.

36. Wölfel, R., Corman, V.M., Guggemos, W., Seilmaier, M., Zange, S., Müller, M.A., Niemeyer, D., Jones, T.C., Vollmar, P., Rothe, C., et al. (2020). Virological assessment of hospitalized patients with COVID-2019. Nature *581*, 465–469.

37. Mammoth Biosciences. (2020). SARS-CoV-2 RNA DETECTR Reagent Kit. https://www.fda.gov/media/141765/download.

38. Sherlock Biosciences. (2020). Instructions for use: SherlockTM CRISPR SARS-CoV-2 kit. https://www.fda.gov/media/137746/download.

39. Jia, N., Jones, R., Yang, G., Ouerfelli, O., and Patel, D.J. (2019). CRISPR-Cas III-A Csm6 CARF Domain Is a Ring Nuclease Triggering Stepwise cA_4 Cleavage with ApA>p Formation Terminating RNase Activity. Mol. Cell *75*, 944–956.e6.

40. Yan, W.X., Hunnewell, P., Alfonse, L.E., Carte, J.M., Keston-Smith, E., Sothiselvam, S., Garrity, A.J., Chong, S., Makarova, K.S., Koonin, E.V., et al. (2019). Functionally diverse type V CRISPR-Cas systems. Science *363*, 88–91.

41. Sofos, N., Feng, M., Stella, S., Pape, T., Fuglsang, A., Lin, J., Huang, Q., Li, Y., She, Q., and Montoya, G. (2020). Structures of the Cmr-β Complex Reveal the Regulation of the Immunity Mechanism of Type III-B CRISPR-Cas. Mol. Cell *79*, 741–757.e7.

42. Elmore, J.R., Sheppard, N.F., Ramia, N., Deighan, T., Li, H., Terns, R.M., and Terns, M.P. (2016). Bipartite recognition of target RNAs activates DNA cleavage by the Type III-B CRISPR-Cas system. Genes Dev. *30*, 447–459.

43. Zhang, J., Graham, S., Tello, A., Liu, H., and White, M.F. (2016). Multiple nucleic acid cleavage modes in divergent type III CRISPR systems. Nucleic Acids Res. *44*, 1789–1799.

44. Makarova, K.S., Timinskas, A., Wolf, Y.I., Gussow, A.B., Siksnys, V., Venclovas, Č., and Koonin, E.V. (2020). Evolutionary and functional classification of the CARF domain superfamily, key sensors in prokaryotic antivirus defense. Nucleic Acids Res. *48*, 8828–8847.

45. Liu, T.Y., Liu, J.J., Aditham, A.J., Nogales, E., and Doudna, J.A. (2019). Target preference of Type III-A CRISPR-Cas complexes at the transcription bubble. Nat. Commun. *10*, 3001.

46. Rambaut, A., Holmes, E.C., O'Toole, Á., Hill, V., McCrone, J.T., Ruis, C., du Plessis, L., and Pybus, O.G. (2020). A dynamic nomenclature proposal for SARS-CoV-2 lineages to assist genomic epidemiology. Nat. Microbiol. *5*, 1403–1407.

47. Loman, N.J., Rowe, W., and Rambaut, A. (2020). NCoV-2019 Novel Coronavirus Bioinformatics Protocol. https://artic.network/ncov-2019/ncov2019-bioinformatics-sop.html.

48. Katoh, K., and Standley, D.M. (2013). MAFFT multiple sequence alignment software version 7: improvements in performance and usability. Mol. Biol. Evol. *30*, 772–780.

49. Nemudryi, A., Nemudraia, A., Wiegand, T., Nichols, J., Deann, T., Hedges, J.F., Cicha, C., Lee, H., Vanderwood, K.K., Bimczok, D., et al. (2021). SARS-CoV-2 genomic surveillance identifies naturally occurring truncations of ORF7a that limit immune suppression. medRxiv. https://doi.org/10.1101/2021.02.22.21252253.

50. Grubaugh, N.D., Gangavarapu, K., Quick, J., Matteson, N.L., De Jesus, J.G., Main, B.J., Tan, A.L., Paul, L.M., Brackney, D.E., Grewal, S., et al. (2019). An amplicon-based sequencing framework for accurately measuring intrahost virus diversity using PrimalSeq and iVar. Genome Biol. *20*, 8.

51. Tyson, J.R., James, P., Stoddart, D., Sparks, N., Wickenhagen, A., Hall, G., Choi, J.H., Lapointe, H., Kamelian, K., Smith, A.D., et al. (2020). Improvements to the ARTIC multiplex PCR method for SARS-CoV-2 genome sequencing using nanopore. bioRxiv. https://doi.org/10.1101/2020.09.04.283077.

CellPress
OPEN ACCESS

STAR★METHODS

KEY RESOURCES TABLE

REAGENT or RESOURCE	SOURCE	IDENTIFIER
Bacterial and virus strains		
E. coli: Bl21 DE3 competent cells	NEB	Cat# C2527I
E. coli: DH5α competent cells	Thermo Fisher Scientific	Cat# 18265017
Biological samples		
SARS-CoV-2	The National Institute of Standards and Technology	Cat# RGTM 10169
SARS-CoV-1	American Type Culture Collection (ATCC)	Cat# VR-3280SD
MERS-CoV	ATCC	Cat# VR-3248SD
Human coronavirus HKU1	ATCC	Cat# VR-3262SD
Influenza B	ATCC	Cat# VR-1885DQ
Human coronavirus NL63	ATCC	Cat# VR-3263SD
Human respiratory syncytial virus	ATCC	Cat# VR-1580DQ
Pseudomonas aeruginosa	ATCC	Cat# 27853D-5
Candida albicans	ATCC	Cat# 10231D-5
Human Nasopharyngeal Swab RNA (Negative and Positive for SARS-CoV-2)	This paper	N/A
Chemicals, peptides, and recombinant proteins		
TCEP	Soltec	Cat# M115
Protease inhibitor cocktail	Thermo Fisher Scientific	Cat# 1861278
MEGAscript T7 transcription kit	Thermo Fisher Scientific	Cat# AMB13345
SUMO protease	Wiedenheft lab	N/A
ATP	Thermo Fisher Scientific	Cat# R0441
Murine RNase Inhibitor	NEB	Cat# M0314
Calcein	MP Biomedical	Cat# 02190167-CF
WarmStart Bst 2.0	NEB	Cat# M0538L
WarmStart RTx Reverse Transcriptase	NEB	Cat# M0380L
Hi-T7 RNA Polymerase	NEB	Cat# M0658
TaqPath 1-Step RT-qPCR Master Mix	Thermo Fisher Scientific	Cat# A15299
SuperScript IV	Thermo Fisher Scientific	Cat# 18090010
Cyclic tetraadenylate	Biolog	Cat# C 335-005
Q5 DNA Polymerase	NEB	Cat# M0491L
NEBNext Ultra II End Repair/dA-Tailing Module	NEB	Cat# E7546
Native Barcoding Expansion 1-12	Oxford Nanopore	Cat# EXP-NBD104
Native Barcoding Expansion 13-24	Oxford Nanopore	Cat# EXP-NBD114
Ligation sequencing kit	Oxford Nanopore	Cat# SQK-LSK109
WarmStart Colorimetric LAMP Master Mix	NEB	Cat# M1800L
Deposited data		
B.1.1.7 lineage SARS-CoV-2 genome sequence 1 (hCoV-19/USA/MT-BHDH-227/2021)	This paper	GISAID ID: EPI_ISL_1081321; GenBank: MW940884
B.1.1.7 lineage SARS-CoV-2 genome sequence 2 (hCoV-19/USA/MT-BHDH-228/2021)	This paper	GISAID ID: EPI_ISL_1081322; GenBank: MW940885

(*Continued on next page*)

Continued

REAGENT or RESOURCE	SOURCE	IDENTIFIER
B.1.1.7 lineage SARS-CoV-2 genome sequence 3 (hCoV-19/USA/MT-BHDH-229/2021)	This paper	GISAID ID: EPI_ISL_1081323; GenBank: MW940886
Recombinant DNA		
Plasmid: pCDF-5xT7-TtCsm	Liu et al.[45]	RRID: Addgene_128572
Plasmid: pCDF-5xT7-TtCsm$^{Csm3\text{-}D34A}$	This paper	N/A
Plasmid: pACYC-TtCas6-4xcrRNA4.5	Liu et al.[45]	RRID: Addgene_127764
Plasmid: pACYC-TtCas6-4xgCoV2N1	This paper	N/A
Plasmid: pRSF-TtCas6	This paper	N/A
Plasmid: pC0075 TtCsm6 His6-TwinStrep-SUMO-BsaI	Gootenberg et al.[14]	RRID: Addgene_115270
Software and algorithms		
Prism 8	GraphPad	https://www.graphpad.com/scientific-software/prism
MinKNOW	Oxford Nanopore	https://community.nanoporetech.com/sso/login?next_url=%2Fprotocols%2Fexperiment-companion-minknow%2Fv%2FMKE_1013_v1_revBM_11Apr2016
Automated SARS-CoV-2 lineage assigner	Rambaut et al.[46]	https://github.com/cov-lineages/pangolin
ARTIC bioinformatic pipeline	Loman et al.[47]	https://artic.network/ncov-2019/ncov2019-bioinformatics-sop.html
Other		
Spin concentrators	Corning	Cat# 431491
HisTrap HP resin	Cytiva	Cat# 17524701
HiLoad Superdex 200 26/600 pg	Cytiva	Cat# 28989336
Superdex 6 Increase 10/300 GL	Cytiva	Cat# 29091596
Microspin G25 columns	Cytiva	Cat# 27-5325-01
StrepTrap HP resin	Cytiva	Cat# 28907546
HisPur Ni-NTA magnetic beads	Thermo Fisher Scientific	Cat# 88832
QIAamp Viral RNA Mini Kit	QIAGEN	Cat# 52906

RESOURCE AVAILABILITY

Lead contact

Further information and requests for resources and reagents should be directed to and will be fulfilled by the leader contact, Blake Wiedenheft (bwiedenheft@gmail.com).

Materials availability

Plasmids generated in this study are available upon request.

Data and code availability

Three sequenced SARS-CoV-2 genomes have been uploaded to GISAID (IDs EPI_ISL_1081321, EPI_ISL_1081322, EPI_-ISL_1081323) and GenBank: MW940884, MW940885, MW940886. Genome IDs are listed in the Key resources table.

The script used to design TtCsm guide RNAs specific to SARS-CoV-2 genomes is available at https://github.com/WiedenheftLab/Type-III_crRNA_Design.

EXPERIMENTAL MODEL AND SUBJECT DETAILS

Escherichia coli DH5α (Thermo Fisher Scientific) cells were used to amplify plasmids used in this paper. *E. coli* BL21 DE3 (NEB) cells were used to express proteins used in this paper. *E. coli* were grown in LB (Lennox) media at either 37°C, or 16°C after induction of protein expression with 0.5 mM IPTG (isopropyl-β-D-thiogalactoside).

METHOD DETAILS

Nucleic acid preparation

Previously published LAMP primers (Eurofins) were designed to amplify the SARS-CoV-2 N-gene.[13] Target SARS-CoV-2 and SARS-CoV-1 RNAs were *in vitro* transcribed with MEGAscript T7 (Thermo Fisher Scientific) from PCR products generated from pairs of synthesized overlapping DNA oligos or using SARS-CoV-2 genome as a template (Table S3) (Eurofins). Previously designed primer pools (IDT) were used for RT-PCR and sequencing of SARS-CoV-2 genomes (https://artic.network/ncov-2019/ncov2019-bioinformatics-sop.html). Transcribed RNAs were purified by denaturing PAGE. Fluorescent reporter RNA A and fluorescent reporter RNA B purified by RNase-free HPLC (Table S2) (IDT). Purified genomes of viral, bacterial and fungal pathogens were used as is, or resuspended in 1x TE (10 mM Tris-HCl pH 7.5, 1 mM Ethylenediaminetetraacetic acid (EDTA)) to ~1x10^6 genomes/μL (Table S5).

Plasmids

Expression vectors for *Thermus thermophilus* type III-A *csm1-csm5* genes, pCDF-5xT7-TtCsm were purchased from Addgene (plasmid # 128572).[45] pCDF-5xT7-TtCsm was used as a template for site-directed mutagenesis to mutate the Csm3 residue D33 to alanine (D33A) to inactivate Csm3-mediated cleavage of target RNA (pCDF-5xT7-TtCsm$^{Csm3\text{-}D34A}$).[29] The CRISPR array in pACYC-TtCas6-4xcrRNA4.5 (Addgene plasmid # 127764)[45] was replaced with a synthetic CRISPR array (GeneArt) containing five repeats and four identical spacers, designed to target the N-gene of SARS-CoV2 (i.e., pACYC-TtCas6-4xgCoV2N1). TtCas6 was PCR amplified from the pACYC-TtCas6-4xcrRNA4.5 plasmid and cloned between the NcoI and XhoI sites of pRSF-1b (MilliporeSigma) (pRSF-TtCas6). The CARF-HEPN nuclease TtCsm6 was expressed from pC0075 TtCsm6 His6-TwinStrep-SUMO-BsaI (Addgene plasmid # 115270).[14]

Protein purifications

Expression and purification of the TtCsm complex was performed as previously described with minor modifications.[45] Briefly, the crRNA plasmid (e.g., pACYC-TtCas6-4xgCoV2N1) was co-transformed with pRSF-TtCas6 and either pCDF-5xT7-TtCsm or pCDF-5xT7-TtCsm$^{Csm3\text{-}D34A}$ into *Escherichia coli* BL21(DE3) cells and grown in LB Broth (Lennox) (Thermo Fisher Scientific) at 37°C to an OD_{600} of 0.5. Cultures were then induced with 0.5 mM IPTG (isopropyl-β-D-thiogalactoside) for expression overnight at 25°C. Cells were pelleted (3,000 × g for 25 mins at 4°C) and lysed via sonication in Lysis buffer (25 mM HEPES pH 7.5, 150 mM KCl, 10 mM imidazole, 1 mM TCEP, 0.01 % Triton X-100, 5 % glycerol, 1 mM PMSF). Lysate was clarified by centrifugation at 10,000 × g for 25 mins at 4°C. The lysate was then heat-treated at 55°C for 45 minutes and further clarified by centrifugation at 10,000 × g for 25 mins at 4°C. His-tagged Csm1 and TtCsm complex were bound to HisTrap HP resin (Cytiva) and washed with Wash buffer (50 mM HEPES pH 7.5, 150 mM KCl, 1 mM TCEP, 5 % glycerol, 20 mM imidazole). Protein was eluted in Lysis buffer supplemented with 300 mM imidazole. Eluted protein was concentrated (100k MWCO Corning Spin-X concentrators) at 4°C before further purification over HiLoad Superdex 200 26/600 or Superose 6 Increase 10/300 GL size-exclusion columns (Cytiva) in 25 mM HEPES pH 7.5, 150 mM NaCl, 5% glycerol, 1 mM TCEP. Fractions containing the TtCsm complex were pooled, concentrated, aliquoted, flash frozen in liquid nitrogen, and stored at −80°C.

Expression and purification of TtCsm6 was performed as previously described with minor modifications.[14] pTtCsm6 was transformed into *Escherichia coli* BL21(DE3) cells and grown in LB Broth (Lennox) (Thermo Fisher Scientific) at 37°C to an OD_{600} of 0.5. Cultures were then incubated on ice for 1 hour, and then induced with 0.5 mM IPTG for expression overnight at 16°C. Cells were lysed via sonication in TtCsm6 Lysis buffer (20 mM Tris-HCl pH 8, 500 mM NaCl, 1 mM TCEP) and lysate was clarified by centrifugation at 10,000 × g for 25 mins at 4°C. The lysate was heat-treated at 55°C for 45 minutes and clarified by centrifugation at 10,000 × g for 25 mins at 4°C. His6-TwinStrep-tagged TtCsm6 was bound to StrepTrap HP resin (Cytiva) and washed in TtCsm6 Lysis buffer. The protein was eluted with TtCsm6 Lysis buffer supplemented with 2.5 mM desthiobiotin and concentrated (10k MWCO Corning Spin-X concentrators) at 4°C. Affinity tags were removed from TtCsm6 using SUMO protease (100 μL of 2.5 mg/ml protease per 20 mg of TtCsm6 substrate) during dialysis against SUMO digest buffer (30 mM Tris-HCl pH 8, 500 mM NaCl 1 mM DTT, 0.15% Igepal) at 4°C overnight. Cleaved His6-TwinStrep tag and uncleaved His6-TwinStrep-TtCsm6 were removed by binding to HisTrap HP resin (Cytiva), and the flow-through was concentrated using Corning Spin-X concentrators at 4°C. Finally, TtCsm6 was purified using a HiLoad Superdex 200 26/600 size-exclusion column (Cytiva) in 20 mM Tris-HCl pH 7.5, 1 mM DTT, 400 mM monopotassium glutamate, 5 % glycerol. Fractions containing TtCsm6 were pooled, concentrated, aliquoted, flash frozen in liquid nitrogen, and stored at −80°C.

To screen guide RNAs in a high throughput format, ten TtCsm complexes were first crudely purified. 8 mL cultures of *E. coli* BL21-DE3 cells transformed with pTtCsm and pT7-5xCRISPR-Cas6 were grown at 37°C and 250 RPM in LB media with selective antibiotics until they reached an OD_{600} reading of 0.4. Protein expression was then induced with the addition of 0.5 mM IPTG to the media, and cells were grown overnight at 16°C. Cells were collected by centrifugation at 4000 RPM, and cell pellets were resuspended in 250 μL of Ni-NTA Equilibration buffer (PBS; 100mM sodium phosphate, 600mM sodium chloride), 0.05% Tween-20 Detergent, 30mM imidazole; pH 8.0). Resuspended cells were sonicated twice for twenty seconds, then clarified by centrifugation at 15,000 rpm for 20 minutes at −4°C to remove cellular debris. The lysate was then heat-treated at 55°C for 45 minutes, and re-clarified by centrifugation at 15,000 rpm, for 30 mins at 4°C. TtCsm was then purified using HisPur Ni-NTA magnetic beads (Thermo Fisher Scientific) according to the manufacturers recommendations, but with modified wash (25 mM HEPES pH 7.5, 150 mM NaCl, 0.05%

Tween-20, 1 mM TCEP) and equilibration (25 mM HEPES pH 7.5, 150 mM NaCl, 1 mM TCEP) and elution buffers (25 mM HEPES pH 7.5, 150 mM NaCl, 1 mM TCEP, 300 mM Imidazole). TtCsm complex concentration was quantified on a Nanodrop (Thermo Fisher Scientific).

Type III CRISPR-based RNA detection

For experiments shown in Figure 1C, RNA was extracted from nasopharyngeal swabs derived from patients that tested negative for SARS-CoV-2 as determined by RT-qPCR. This RNA was used as is or spiked with *in vitro* transcribed SARS-CoV-2 or SARS-CoV-1 RNA. These RNA samples were mixed with 250 μM ATP, 500 nM fluorescent reporter RNA A, 500 nM TtCsm complex, and 2500 nM of TtCsm6 in reaction buffer (20 mM Tris-HCl pH 7.9, 200 mM Monopotassium glutamate, 10 mM Ammonium sulfate, 5 mM Magnesium sulfate and 1 mM TCEP (tris(2-carboxyethyl)phosphine)) in a 30 μL reaction. Reactions were incubated at 60°C (CRISPR-Csm alone), and fluorescence was measured over time in an ABI 7500 Fast Real-Time PCR System (Applied Biosystems), using the manufacturers default filter settings for FAM dye. Fluorescence measurements at an incubation time of 45 minutes are reported.

For experiments shown in Figure 2C, an RNA detection mixture was made containing either 25 nM $TtCsm^{Csm3\text{-}D34A}$ N1, or 25 nM $TtCsm^{Csm3\text{-}D34A}$ N9, or 2.5 nM each of ten complexes ($TtCsm^{Csm3\text{-}D34A}$ N1, N3, N6, N7, N8, N9, N10, N11, N12 and I1), mixed with 250 μM ATP, 150 nM fluorescent reporter RNA B, 300 nM TtCsm6, in reaction buffer (20 mM Tris-HCl pH 7.8, 250 mM Monopotassium glutamate, 10 mM Ammonium sulfate, 5 mM Magnesium sulfate and 1 mM TCEP). 3 μL of RNA extracted from a patient nasopharyngeal swab with high SARS-CoV-2 viral load (~$5x10^8$ copies/μL) was added to 27 μL of the above RNA detection mixture. Alternatively, RNA from this positive patient sample was first diluted 10– or 100–fold into RNA extracted from a patient negative for SARS-CoV-2 (CT > 40), and 3 μL of these dilutions was added to 27 μL of the above RNA detection mixture. Reactions were incubated at 60°C and fluorescence was measured every 10 s for up to 20 minutes, in a QuantStudio 3 Real-Time PCR system (ThermoFisher), using the manufacturers default filter settings for FAM dye.

Colorimetric CRISPR-Csm based detection

$TtCsm^{Csm3\text{-}D34A}$ stocks were buffer exchanged into a low buffering capacity buffer (0.5 mM Tris-HCl pH 8.8, 50 mM Potassium chloride, 10 mM Ammonium sulfate, 8 mM Magnesium sulfate) using Microspin G25 columns (Cytiva) as per the manufacturer's instructions. TE buffer (10 mM Tris-HCl pH 7.5, 1 mM EDTA) or *in vitro* transcribed SARS-CoV-2 or SARS-CoV-1 RNA were incubated with 200 nM $TtCsm^{Csm3\text{-}D34A}$ in 1x WarmStart Colorimetric LAMP Master Mix (NEB), supplemented with an additional 1 mM ATP, in a 25 μL reaction. The volume of buffer-exchanged TtCsm used contributed approximately 40 μM Tris-HCl pH 8.8 buffer to the final reaction. Reactions were assembled on ice and imaged on an LED tracing pad with a Galaxy S9 phone (Samsung). Then reactions were incubated at 60°C for 30 minutes, rapidly cooled, and imaged again.

Visible fluorometric CRISPR-Csm based detection

TE buffer or *in vitro* transcribed SARS-CoV-2 or SARS-CoV-1 RNA were incubated with 500 nM $TtCsm^{Csm3\text{-}D34A}$ in reaction buffer (20 mM Tris-HCl pH 8.8, 100 mM Potassium chloride, 10 mM Ammonium sulfate, 6 mM Magnesium sulfate, 0.5 mM Manganese chloride, 1 mM TCEP, 1 mM ATP and 25 μM Calcein), in a 30 μL reaction. Reactions were incubated at 60°C, and fluorescence was measured over time in an ABI 7500 Fast Real-Time PCR System (Applied Biosystems), using the manufacturers default filter settings for FAM dye. After incubating at 60°C for 50 minutes, the same reactions were then imaged under visible light, and under UV light (365 nm) with a Galaxy S9 phone (Samsung). To screen guide RNAs in a high throughput format (Figure 2B), 200 nM crude purified TtCsm complex was incubated with 10^{12} copies of IVT SARS-CoV-2 RNA in the above buffers and fluorescence was recorded in a ABI 7500 Fast Real-Time PCR System (Applied Biosystems) machine as above.

RT-LAMP-T7-Csm

Isothermal amplification of nucleic acids in swab samples was performed by RT-LAMP. In brief, 25 μL reactions contained 8 units (U) of WarmStart Bst 2.0 (NEB), and 7.5 U of WarmStart RTx Reverse Transcriptase (NEB), 1.4 mM dNTPs, LAMP primers, 25 U of Murine RNase Inhibitor (NEB) in reaction buffer (20 mM Tris-HCl pH 7.8, 8 mM Magnesium sulfate, 10 mM Ammonium sulfate, 50 mM potassium chloride, 0.1% Tween-20). LAMP primers designed to amplify the SARS-CoV-2 N-gene,[13] were added at an optimized final concentration of 0.2 μM F3 and B3, 0.4 μM LoopF and LoopB, 1.6 μM BIP, 0.53 μM FIP, and 1.07 μM of T7-FIP (Table S4). The T7-FIP primer consists of a T7 promoter fused to the 5¿ end of the FIP primer, and allows for the generation of T7 transcription templates during the second step of T7-Csm reaction. RT-LAMP reactions were performed using 5 μL of input RNA at 65°C for 29 minutes. 3 μL of RT-LAMP reactions were mixed with 27 μL of a modified T7-Csm fluorescent detection reaction containing 0.5 mM rNTPs, 300 nM TtCsm6, 5.5 units of Hi-T7 RNA Polymerase (NEB), 150 nM fluorescent reporter RNA B, and 20 nM of either $TtCsm^{Csm3\text{-}D34A}$ N1 or N9, in reaction buffer (40 mM Tris-HCl pH 7.5, 4 mM Magnesium chloride, 50 mM Sodium chloride, 2 mM spermidine, 1 mM DTT). Reactions were incubated at 55°C for up to 20 min and fluorescence kinetics was monitored in a QuantStudio 3 Real-Time PCR system (ThermoFisher) as described above.

LoD standards were prepared by diluting SARS-CoV-2 RNA into RNA extracted from COVID-19-negative patient nasopharyngeal swabs. Concentrations were determined with RT-qPCR using a standard curve generated from 10-fold dilution series ($1x10^6$-$1x10^\circ$) of IVT fragment.

CellPress
OPEN ACCESS

Human clinical sample collection and preparation

Nasopharyngeal swabs from patients that either tested negative or positive for SARS-CoV-2 were collected in viral transport media. RNA was extracted from all patient samples using QIAamp Viral RNA Mini Kit (QIAGEN).

RT-qPCR

RT-qPCR was performed using two primers pairs (N1 and N2) and probes from the 2019-nCoV CDC EUA Kit (IDT#10006606). SARS-CoV-2 in RNA-extracted, nasopharyngeal patient samples was detected and quantified using one-step RT-qPCR in ABI 7500 Fast Real-Time PCR System according to CDC guidelines and protocols (https://www.fda.gov/media/134922/download). In brief, 20 μL reactions included 8.5 μL of Nuclease-free Water, 1.5 μL of Primer and Probe mix (IDT, 10006713), 5 μL of TaqPath 1-Step RT-qPCR Master Mix (ThermoFisher, A15299) and 5 μL of the template. Nuclease-free water was used as negative template control (NTC). Amplification was performed as follows: 25°C for 2 min, 50°C for 15 min, 95°C for 2 min followed by 45 cycles of 95°C for 3 s and 55°C for 30 s. To quantify viral genome copy numbers in the samples, standard curves for N1 and N2 were generated using a dilution series of a SARS-CoV-2 synthetic RNA fragment (RTGM 10169, National Institute of Standards and Technology) spanning N gene with concentrations ranging from 10 to 10^6 copies per μL. Three technical replicates were performed at each dilution. The NTC showed no amplification throughout the 45 cycles of qPCR.

Bioinformatic design of TtCsm crRNA guides targeting SARS-CoV-2

An alignment of 45,641 SARS-CoV-2 genomes was downloaded from the GISAID database (Global Initiative for Sharing All Influenza Data; https://www.gisaid.org/ on 6-23-2020.[34,48] The alignment was scanned for conservation with a 40-nucleotide sliding window, and 40-nucleotide segments with strong conservation were saved for downstream analysis. Next, four nucleotides flanking the above 40-nucleotide candidate viral target sequences were checked for base pairing to the first four nucleotides of the prospective 5′-crRNA handle (underlined; 5′-AUUG<u>CGAC</u>-3′), only candidates lacking handle complementarity were considered further. Candidate sites with less than two mismatches to SARS-CoV (NC_004718.3) and MERS-CoV (NC_019843.3) in the first 18 nucleotides of the target sequence were discarded. Next, candidate crRNAs targeting the above sites were screened for potential cross-reactivity with human mRNAs and a list of human pathogens and common respiratory flora downloaded from the FDA's Emergency Use Authorization requirements (downloaded on 7-29-2020) using BLAST (E-value 1000). The remaining 6,229 crRNA sequences were then sorted by genomic location and only guides that were located 3′ of the SARS-CoV-2 ORF3a gene (positions 25,393 to 29,903) were considered further. Finally, 76 guides were selected from the remaining pool that had the greatest conservation among SARS-CoV-2 sequences and the largest number of mismatches to SARS-CoV and MERS-CoV sequences.

Sequencing of SARS-CoV-2 RNA isolated from patient samples

SARS-CoV-2 genomic RNA isolated from patient samples was sequenced as previously described.[49] In brief, 10 μL of SARS-CoV-2 genomic RNA extracted from nasopharyngeal patient swabs was first reverse transcribed with SuperScript IV (ThermoFisher) according to the manufacturer's instructions. The ARTIC Network protocol was followed to generate a sequence amplicon library covering the whole SARS-CoV-2 genome on Oxford Nanopore using a ligation sequencing kit (Oxford Nanopore, SQK-LSK109) (https://artic.network/ncov-2019/ncov2019-bioinformatics-sop.html).[50,51] Two multiplex PCR reactions were performed with primer pools described in the ARTIC nCoV-2019 V3 Panel (Table S6), amplified with Q5 DNA Polymerase (NEB). The two resulting amplicon pools for each patient sample were then combined and used for library preparation. Samples were end repaired (NEB, E7546) and then barcoded using Native Barcoding Expansion Kits (Oxford Nanopore, EXP-NBD104 and EXP-NBD114). Barcoded samples were pooled together and then Nanopore adaptors were ligated.

The multiplexed library was loaded onto the MinION flowcell, and a total of 0.3 Gb of raw sequencing data was collected per patient sample. Raw Nanopore reads were base-called in high-accuracy mode (Oxford Nanopore, MinKNOW), and further analyzed using the ARTIC bioinformatic pipeline for COVID-19 (https://artic.network/ncov-2019/ncov2019-bioinformatics-sop.html).[47] Consensus sequences were uploaded to GISAID (https://www.gisaid.org/), IDs: EPI_ISL_1081321, EPI-ISL_1081322, EPI_ISL_1081323.[34] These three SARS-CoV-2 genome sequences were identified as members of the B.1.1.7 lineage by an automated lineage assigner[46] (https://github.com/cov-lineages/pangolin).

QUANTIFICATION AND STATISTICAL ANALYSIS

All experiments were performed in triplicate or duplicate and error is reported as ± 1 standard deviation. The merged datasets of replicates of fluorescence kinetics of direct Csm-based detection of SARS-CoV-2 RNA in patient samples was fit to a simple linear regression, in Prism 9 (Graphpad). The fitted slopes of SARS-CoV-2 RNA-containing patient samples were compared pairwise to the negative swab RNA control by an F-test, ****$p < 0.0001$.

Cell Reports Medicine, Volume *2*

Supplemental information

Intrinsic signal amplification by type III CRISPR-Cas systems provides a sequence-specific SARS-CoV-2 diagnostic

Andrew Santiago-Frangos, Laina N. Hall, Anna Nemudraia, Artem Nemudryi, Pushya Krishna, Tanner Wiegand, Royce A. Wilkinson, Deann T. Snyder, Jodi F. Hedges, Calvin Cicha, Helen H. Lee, Ava Graham, Mark A. Jutila, Matthew P. Taylor, and Blake Wiedenheft

Supplementary Information

Intrinsic Signal Amplification by Type-III CRISPR-Cas Systems Provides a Sequence-Specific Viral Diagnostic

Andrew Santiago-Frangos, Laina N. Hall, Anna Nemudraia, Artem Nemudryi, Pushya Krishna, Tanner Wiegand, Royce A. Wilkinson, Deann T. Snyder, Jodi F. Hedges, Calvin Cicha, Helen H. Lee, Ava Graham, Mark A. Jutila, Matthew P. Taylor, and Blake Wiedenheft*

Department of Microbiology and Immunology, Montana State University, Bozeman, MT 59717, USA

*Correspondence to BW: bwiedenheft@gmail.com

Supplementary Fig. 1

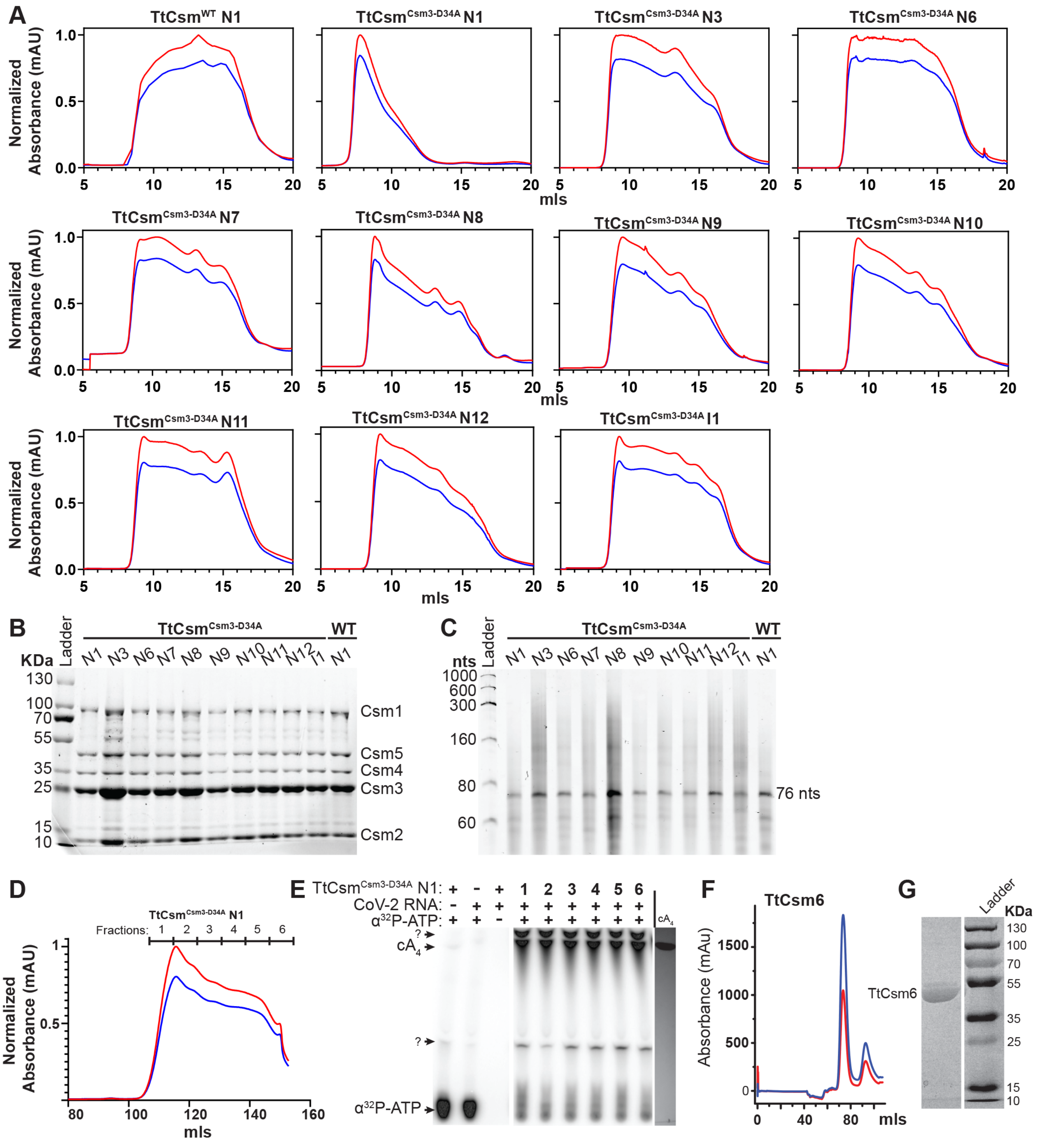

Figure S1. Purification of TtCsm complexes and TtCsm6 nuclease. Related to Figures 1-3. (**A**) Size exclusion chromatography (SEC) profiles of TtCsm^{WT} and TtCsm$^{Csm3\text{-}D34A}$ complexes loaded with different crRNA guides. SEC performed using a Superose 6 Increase 10/300 GL size-exclusion column (Cytiva). Normalized absorbance (mAU) was measured at 260nm (red) and 280nm (blue). Fractions 9 up to16 of each SEC purification were collected, concentrated, and stored at -80°C. (**B**) The fractions were combined, concentrated and run on an SDS-PAGE. All five Csm proteins are present and the intensities of each band correspond with our understanding of the protein stoichiometry of the assembled TtCsm complex. (**C**) RNA was isolated from the pooled and concentrated SEC fractions. Denaturing urea polyacrylamide gel of nucleic acids associated with each TtCsm complex. The full-length crRNA intermediate is expected to be 76 nucleotides (nts) long. (**D**) SEC profile of TtCsm$^{Csm3\text{-}D34A}$ N1 complex. Six successive fractions, representing the entire peak, were collected concentrated and stored separately. (**E**) Sequence-specific activation of Cas10 was estimated for each of the six fractions in panel D. ^{32}P-ATP polymerization was measured using thin-layer chromatography (TLC). 500 nM TtCsm$^{Csm3\text{-}D34A}$ N1 complex was incubated with 10^{10} copies of target RNA, 50 µM ATP and 10 nM α^{32}P-ATP, at 60°C for 1 hour. Nucleic acids were phenol-chloroform extracted from each reaction and spotted on a silica gel TLC plate coated with fluorescent indicator F254, developed in solvent (0.2 M ammonium bicarbonate pH 9.3, 70% ethanol, 30% water). An unlabeled cA_4 standard (Axxora) was run on the same TLC plate, in a parallel lane, and was visualized by illumination with a handheld shortwave (254 nm) UV lamp (Analytik Jena) and a Galaxy S9 phone (Samsung). One of the two major ^{32}P-labelled products generated by target RNA-bound TtCsm complex migrates similarly to the cA_4 standard. All TtCsm complexes polymerize similar amounts of α^{32}P-ATP (bottom band) into similar ratios of similarly migrating products (top bands). (**F**) SEC profile of TtCsm6 ancillary nuclease purified on a Superdex 200 26/600 size-exclusion column (Cytiva). (**G**) SDS-PAGE gel image of purified TtCsm6.

Supplementary Fig. 2

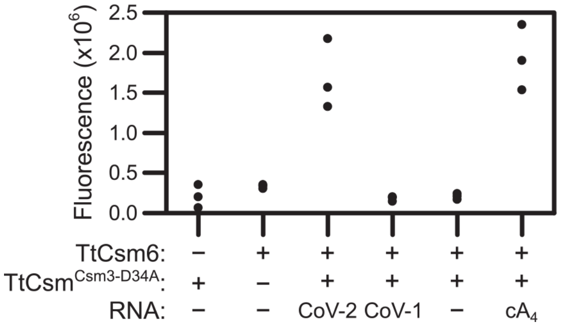

Figure S2. Csm-mediated synthesis of cA_4 is necessary for activation of the non-sequence specific nuclease Csm6. Related to Figure 1. Detection of *in vitro* transcribed SARS-CoV-2 RNA using the type III surveillance complex $TtCsm^{Csm3-D34A}$ and the ancillary nuclease, TtCsm6. $TtCsm^{Csm3-D34A}$, TtCsm6 and SARS-CoV-2 RNA, but not SARS-CoV-1 RNA, are all required to trigger cleavage of the fluorescent RNA reporter. Addition of cA_4, bypasses the signaling pathway and activates TtCsm6 directly, cleaving the reporter RNA in the absence of SARS-CoV-2 RNA (last lane). These reactions were performed using 10^8 copies of *in vitro* transcribed SARS-CoV-1 or -2 RNA spiked into nasopharyngeal swab clinical matrix. Results from three technical replicates are shown. Results presented in the final column (cA4) demonstrate that the florescent signal is generated by cA_4-dependent activation of Csm6.

Supplementary Fig. 3

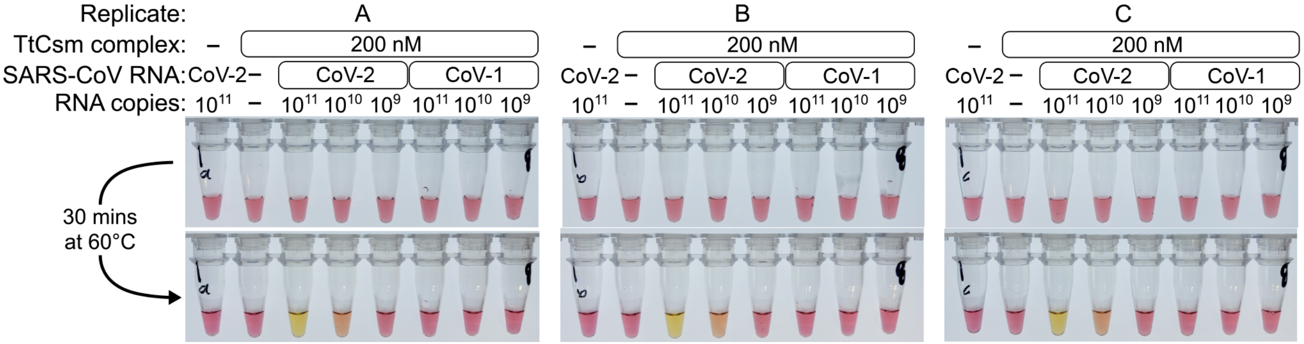

Figure S3. Colorimetric detection of a specific RNA sequence. Related to Figure 1. TtCsm$^{Csm3\text{-}D34A}$ was incubated with either RNA target, in the presence of ATP, for 30 minutes at 60°C. Specific RNA recognition, and associated Cas10-mediated ATP polymerization, causes acidification of the solution, and changes the color of Phenol Red pH indicator from fuchsia, through orange, to yellow. Three technical replicates (A, B and C) are shown.

Supplementary Fig. 4

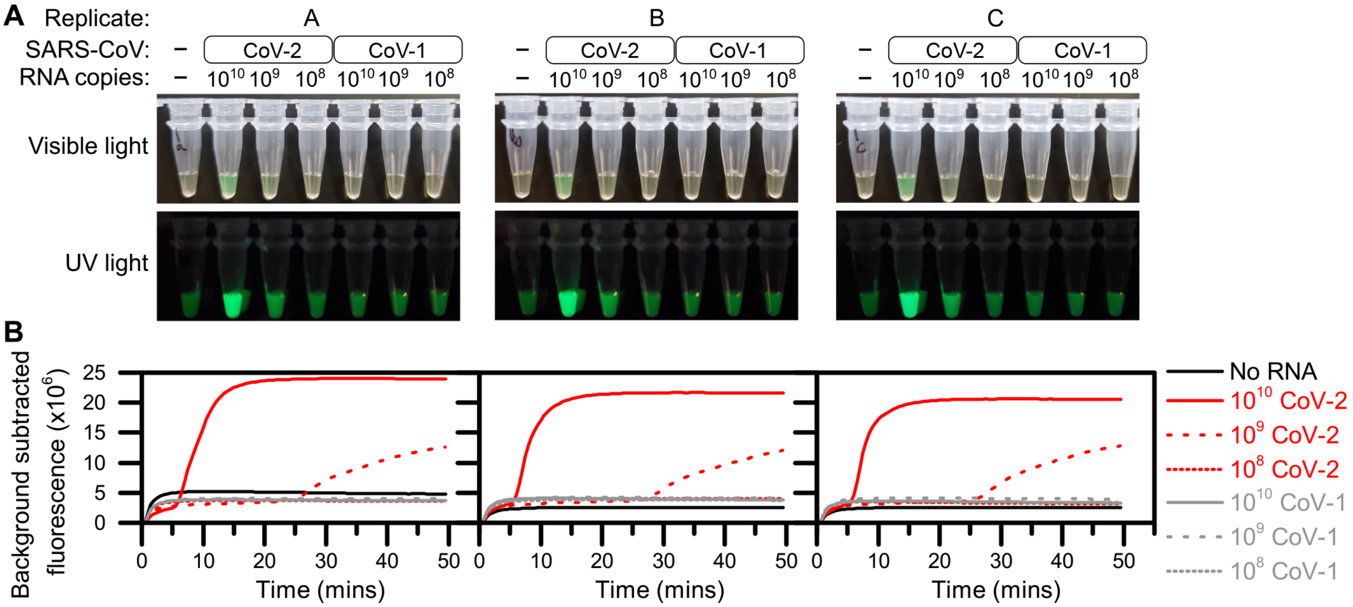

Figure S4. Visible fluorometric detection of a specific RNA sequence. Related to Figure 1. (**A**) TtCsm$^{Csm3\text{-}D34A}$ was incubated with no RNA, a specific (i.e., SARS-CoV-2) or non-specific (SARS-CoV-1) RNA target, in the presence of ATP for 50 minutes at 60°C. The reactions contain an excess of Mg^{2+} relative to Mn^{2+} ions, and the metal indicator Calcein. Calcein preferentially binds Mn^{2+}, forming a quenched complex. Specific RNA recognition, and associated Cas10-mediated ATP polymerization, generates pyrophosphate which precipitates with Mg2+ and Mn2+ ions. Excess Mg^{2+} ions bind Calcein to form a highly fluorescent complex that can be seen by eye in visible light or with UV light. (**B**) Kinetics of Calcein fluorescence for corresponding reactions shown in panel A. 10^{10} SARS-CoV-2 RNAs can be detected in 10 minutes, and 10^{9} copies are detected in 40 minutes. No increase in fluorescence is seen beyond the first five minutes for either a no RNA control, or for SARS-CoV-1 RNA containing samples.

Supplementary Fig. 5

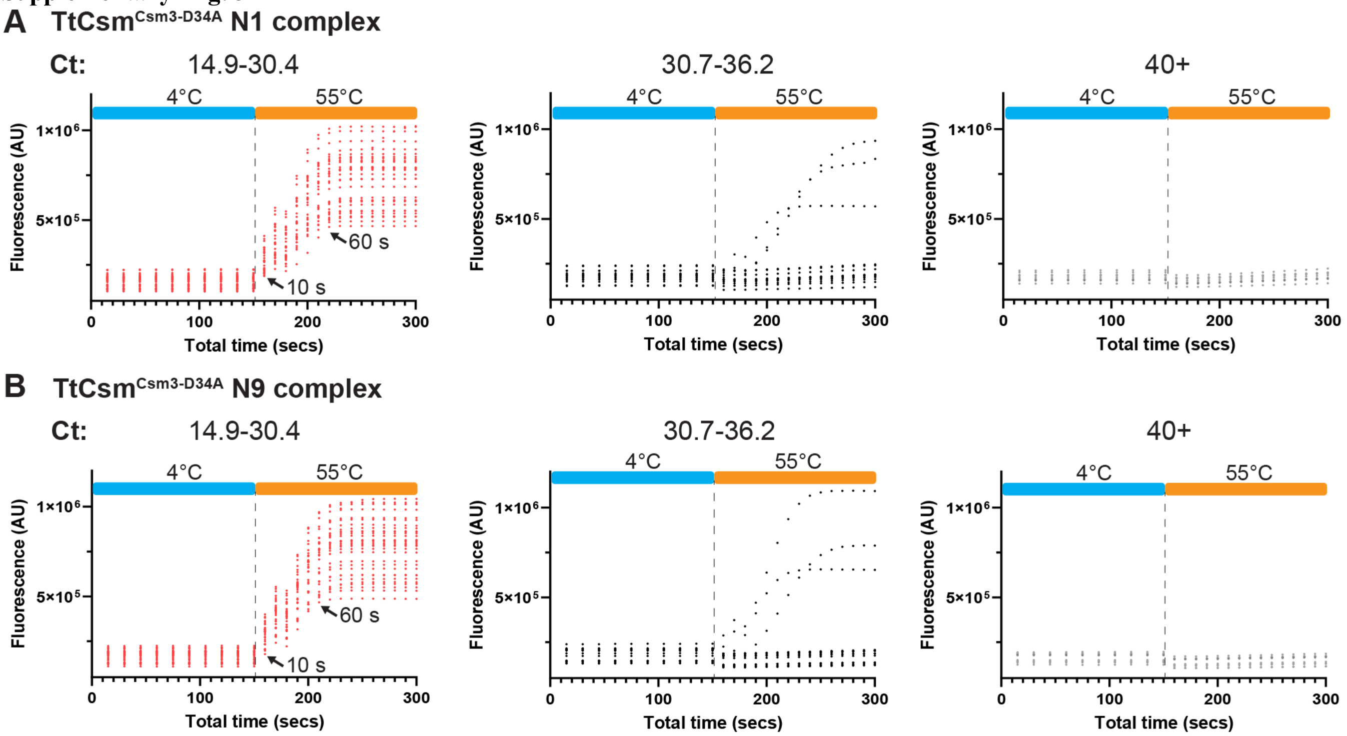

Figure S5. Rapid and specific detection of SARS-CoV-2 by RT-LAMP-T7-Csm. Related to Figure 3. Raw fluorescence kinetics for the T7-Csm stage of RT-LAMP-T7-Csm detection of SARS-CoV-2 RNA from patient samples with Ct values of 14.9-30.4 (left), 30.7-36.2 (middle), and 40+ (right), as detected by the RNase dead TtCsm complex (dTtCsm) loaded with (**A**) crRNA$_{N1}$ or (**B**) crRNA$_{N9}$. Reactions were first incubated in an RT-PCR machine at 4°C and fluorescence was measured every 15 seconds for 150 seconds. Fluorescence readings in both positive and negative reactions are low until the T7-Csm reactions are heated to 55°C, upon which fluorescence rapidly increases within 10 seconds and continues to increase for 60 seconds in most samples.

Supplementary Fig. 6

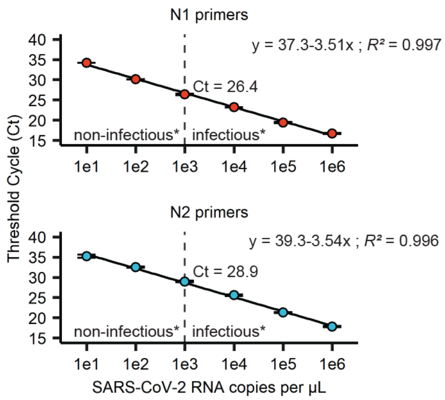

Figure S6. Standard curves for absolute quantification of SARS-CoV-2 titers. Related to Figures 2 and 3. A 10-fold dilution series of SARS-CoV-2 synthetic RNA fragment (RTGM 10169, National Institute of Standards and Technology) containing the Nucleocapsid gene was used. Data was plotted as Cycle Threshold (Ct) on y-axis versus log10 (copies per ml) on x-axis. The mean Ct of three technical replicates are shown, error bars represent ± 1 standard deviation. Trend lines were fit to the data using the geom_smooth function of the ggplot2 R package; linear equations and R2 values are shown. Boundaries corresponding to non-infectious and infectious Ct values refer to observations that patients with viral titers below 10^6 copies/ml are rarely infectious.

Table S1: TtCsm guide RNAs. Related to Figures 1-3 and S1-S5.

Name	***Sequence (5’-3’)*** **(target binding region underlined)**
N1 (76 nt guide)	AUUGCGACACGCUGAAGCGCUGGGGGCAAAUUGUGCAAUUUGCGGCCAGUU GCAAGGGAUUGAGCCCCGUAAGGGG
N3 (76 nt guide)	AUUGCGACGGCCGACGUUGUUUUGAUCGCGCCCCACUGCGUUCUCCAUGUUG CAAGGGAUUGAGCCCCGUAAGGGG
N6 (76 nt guide)	AUUGCGACGUUGCGACUACGUGAUGAGGAACGAGAAGAGGCUUGACUGGUU GCAAGGGAUUGAGCCCCGUAAGGGG
N7 (76 nt guide)	AUUGCGACAGCAGCAGCAAAGCAAGAGCAGCAUCACCGCCAUUGCCAGGUU GCAAGGGAUUGAGCCCCGUAAGGGG
N8 (76 nt guide)	AUUGCGACAUGCUUUAGUGGCAGUACGUUUUUGCCGAGGCUUCUUAGAGUU GCAAGGGAUUGAGCCCCGUAAGGGG
N9 (76 nt guide)	AUUGCGACUUCCGAAGAACGCUGAAGCGCUGGGGGCAAAUUGUGCAAUGUU GCAAGGGAUUGAGCCCCGUAAGGGG
N10 (76 nt guide)	AUUGCGACAUUCAGCAAAAUGACUUGAUCUUUGAAAUUUGGAUCUUUGGUU GCAAGGGAUUGAGCCCCGUAAGGGG
N11 (76 nt guide)	AUUGCGACCAGUUUGCUGUUUCUUCUGUCUCUGCGGUAAGGCUUGAGUGUU GCAAGGGAUUGAGCCCCGUAAGGGG
N12 (76 nt guide)	AUUGCGACGUCAGCACUGCUCAUGGAUUGUUGCAAUUGUUUGGAGAAAGUU GCAAGGGAUUGAGCCCCGUAAGGGG
I1 (76 nt guide)	AUUGCGACAAAAGCGAAAACGUUUAUAUAGCCCAUCUGCCUUGUGUGGGUU GCAAGGGAUUGAGCCCCGUAAGGGG

Table S2: Fluorescent reporter RNAs. Related to Figures 1-3 and S5.

Name	*Sequence (5'-3')*	*Associated figure*
RNA reporter A	/56-FAM/rCrUrCrUrCrU/3IABkFQ/	1
RNA reporter B	/56-FAM/rArUrCrUrUrCrUrUrArU/3IABkFQ/	2 and 3

Table S3: Primers to generate IVT RNA templates. Related to Figures 1, 2, S2, S3 and S4

Primer name	***Sequence (5'-3')***
SARS-CoV-2 N1 T7 template Forward	GATAATACGACTCACTATAGGGAACTGATTACAAACATTGGCCGCAAATTGCACAATT
SARS-CoV-2 N1 T7 template Reverse	GCGCGACATTCCGAAGAACGCTGAAGCGCTGGGGGCAAATTGTGCAATTTGCGGCC
SARS-CoV-1 N1 T7 template Forward	GATAATACGACTCACTATAGGGAACTGATTACAAACATTGGCCGCAAATTGCACAATT
SARS-CoV-1 N1 T7 template Reverse	GCGTGACATTCCAAAGAATGCAGAGGCACTTGGAGCAAATTGTGCAATTTGCGGCC
SARS-CoV-2 target all ten complexes_F	CTAGAGCTCGATAATACGACTCACTATAGGGCGTGTTGTTTTAGATTTCATCTAAACG
SARS-CoV-2 target all ten complexes_R	ATCCTGCAGGCACACTGATTAAAGATTGCTATGTG

Table S4: Primers for RT-LAMP. Related to Figures 3 and S5.

Primer name	***Sequence (5'-3')***
F3	GCTGCTGAGGCTTCTAAG
B3	GCGTCAATATGCTTATTCAGC
BIP	TCAGCGTTCTTCGGAATGTCGCTGTGTAGGTCAACCACG
FIP	GCGGCCAATGTTTGTAATCAGTAGACGTGGTCCAGAACAA
Loop Forward	CCTTGTCTGATTAGTTCCTGGT
Loop Reverse	TGGCATGGAAGTCACACC
T7-FIP	TAATACGACTCACTATAGGGAGACGTGGTCCAGAACAA

Table S5: Purified genomic nucleic acids. Related to Figure 3.

Name	***Source***
SARS-CoV-2	The National Institute of Standards and Technology (RGTM 10169)
SARS-CoV-1	American Type Culture Collection (ATCC) (VR-3280SD)
MERS-CoV	ATCC (VR-3248SD)
Human coronavirus HKU1	ATCC (VR-3262SD)
Influenza B	ATCC (VR-1885DQ)
Human coronavirus NL63	ATCC (VR-3263SD)
Human respiratory syncytial virus	ATCC (VR-1580DQ)
Pseudomonas aeruginosa	ATCC (27853D-5)
Candida albicans	ATCC (10231D-5)
